# Aerobic exercise for Alzheimer's disease: A randomized controlled pilot trial

**DOI:** 10.1371/journal.pone.0170547

**Published:** 2017-02-10

**Authors:** Jill K. Morris, Eric D. Vidoni, David K. Johnson, Angela Van Sciver, Jonathan D. Mahnken, Robyn A. Honea, Heather M. Wilkins, William M. Brooks, Sandra A. Billinger, Russell H. Swerdlow, Jeffrey M. Burns

**Affiliations:** 1 University of Kansas Alzheimer’s Disease Center, Fairway, KS, United States of America; 2 Department of Psychology, University of Kansas, Lawrence, KS, United States of America; 3 Department of Biostatistics, University of Kansas Medical Center, Kansas City, KS, United States of America; 4 Hoglund Brain Imaging Center, University of Kansas Medical Center, Kansas City, KS, United States of America; 5 Department of Physical Therapy and Rehabilitation Science, University of Kansas Medical Center, Kansas City, KS, United States of America; Swinburne University, AUSTRALIA

## Abstract

**Background:**

There is increasing interest in the role of physical exercise as a therapeutic strategy for individuals with Alzheimer’s disease (AD). We assessed the effect of 26 weeks (6 months) of a supervised aerobic exercise program on memory, executive function, functional ability and depression in early AD.

**Methods and findings:**

This study was a 26-week randomized controlled trial comparing the effects of 150 minutes per week of aerobic exercise vs. non-aerobic stretching and toning control intervention in individuals with early AD. A total of 76 well-characterized older adults with probable AD (mean age 72.9 [7.7]) were enrolled and 68 participants completed the study. Exercise was conducted with supervision and monitoring by trained exercise specialists. Neuropsychological tests and surveys were conducted at baseline,13, and 26 weeks to assess memory and executive function composite scores, functional ability (Disability Assessment for Dementia), and depressive symptoms (Cornell Scale for Depression in Dementia). Cardiorespiratory fitness testing and brain MRI was performed at baseline and 26 weeks. Aerobic exercise was associated with a modest gain in functional ability (Disability Assessment for Dementia) compared to individuals in the ST group (X2 = 8.2, p = 0.02). There was no clear effect of intervention on other primary outcome measures of Memory, Executive Function, or depressive symptoms. However, secondary analyses revealed that change in cardiorespiratory fitness was positively correlated with change in memory performance and bilateral hippocampal volume.

**Conclusions:**

Aerobic exercise in early AD is associated with benefits in functional ability. Exercise-related gains in cardiorespiratory fitness were associated with improved memory performance and reduced hippocampal atrophy, suggesting cardiorespiratory fitness gains may be important in driving brain benefits.

**Trial registration:**

ClinicalTrials.gov NCT01128361

## Introduction

An estimated 5.3 million Americans have Alzheimer’s disease (AD) and the prevalence is expected to double by mid-century.[1] There are currently no disease-modifying or preventive treatments for AD.[2] However, animal research suggests that exercise positively impacts brain health through neurotrophic, neurogenic and vascular mechanisms.[3–8] Limited but compelling data suggests that exercise may decrease neuropathological burden[9] and increase hippocampal neurogenesis.[10]

Observational evidence in humans suggests higher levels of cardiorespiratory fitness and physical activity are associated with greater brain volume, less brain atrophy, slower dementia progression and reduced risk of dementia.[11–17] Increased cardiorespiratory fitness also attenuates the detrimental effects of cerebral amyloid on cognition.[18] Randomized controlled trials of aerobic exercise (AEx) in individuals with mild cognitive impairment (MCI)[19] and subjective memory complaints[20] have found exercise improved cognitive function. A separate home-based program of exercise and counseling benefited physical function and depression compared to usual care in persons with AD.[21] Another study of people with MCI showed some cognitive test score improvement when analyses were restricted to compliant exercisers.[22] Although current evidence remains insufficient to conclude that exercise is an effective therapeutic for AD or cognitive decline,[2, 23] exercise continues to be a promising area of research for AD treatment.[24] Aerobic exercise offers a low-cost, low-risk, widely-available intervention that may have disease modifying effects. Demonstrating that aerobic exercise alters the AD process would have enormous public health implications.

Our objective for this pilot study was to establish preliminary efficacy for a community-based, structured aerobic exercise intervention that meets standard public health guidelines (~ 150 minutes / week of moderate intensity aerobic exercise). We hypothesized that cognition, functional ability, and depression would benefit from aerobic exercise compared to non-aerobic stretching and toning exercises in a well-characterized sample of individuals with AD-related cognitive impairment. We also hypothesized that these benefits would be proportional to cardiorespiratory fitness gain.

## Methods

The Alzheimer’s Disease Exercise Program Trial (ADEPT: ClinicalTrials.gov, NCT01128361) was designed as a pilot randomized controlled trial of 26 weeks of aerobic exercise (AEx) versus a non-aerobic stretching and toning control program (ST). The study included adults over 55 in the earliest stages of AD-related cognitive decline. Participants were assigned to AEx and ST groups in a 1:1 ratio. We recruited participants from August 2, 2010 through September 26, 2014. Follow-up testing was completed by April 17, 2015. Details of the study protocol have been previously published.[25] All testing was performed at the University of Kansas Medical Center. The intervention was administered at 16 YMCA of Greater Kansas City facilities.

### Participants

Participants were recruited as a convenience sample of volunteers through print advertising, community talks, memory clinic referral and existing research participant databases. The University of Kansas Alzheimer’s Disease Center (KU ADC) and the YMCA jointly designed and distributed these materials. YMCA membership and certified personal trainer fees were covered by the study. Inclusion criteria included MCI or dementia with etiologic diagnosis of probable AD based on clinical and cognitive test results using standard criteria;[26, 27] Clinical Dementia Rating (CDR) of 0.5 or 1 (very mild to mild dementia);[28] at least 55 years of age; sedentary or underactive as defined by the Telephone Assessment of Physical Activity;[29] community dwelling with a supportive caregiver willing to accompany participants to visits as necessary; adequate visual and auditory ability to perform cognitive testing; stable medication dose (30 days); and ability to participate in all scheduled evaluations and the exercise program. Exclusion criteria included clinically significant psychiatric disorder; systemic illness or infection likely to affect safety; clinically-evident stroke; myocardial infarction or coronary artery disease in the last 2 years; uncontrolled hypertension in the last 6 months; cancer in the last 5 years; drug or alcohol abuse in the last 2 years; insulin dependent diabetes; or significant pain or musculoskeletal symptoms that would prohibit exercise.

Interested individuals completed a telephone screen of medical history followed by in-person screening of those who remained interested and appeared eligible. All participants and a study partner/caregiver provided institutionally approved, written, informed consent under a study protocol (#11969) approved by the KU Medical Center Institutional Review Board, which also acted as the compliance entity for the YMCA. The screening evaluation included a thorough clinical examination including CDR.[28]

### Sample size, randomization, blinding and safety

Participants were block randomized, stratified by age (split at 75) and sex, to balance treatment arms. Our enrollment goal of 80 was determined to provide estimates of intervention effect sizes as well as preliminary hypothesis testing with the recognition that this small pilot study would not be definitive. We reviewed the exercise intervention literature, which has widely varying effect estimates from a diverse participant population with relatively few trials specifically for individuals with cognitive impairment. We thus selected a sample size with 20% expected attrition that would yield 50% power to detect an effect in our primary cognitive outcomes. Additional detail regarding the sample size calculation, including the hypothesis tested, parameters used, and allowances made for refusals and losses to follow-up can be found in our published protocol.[25] One investigator (JDM) constructed the allocation schedule using SAS, placing index cards in 320 sequentially numbered, sealed envelopes grouped by age and sex strata. Envelopes were opened after baseline testing by staff not involved with primary outcome measure testing. Psychometric and cardiorespiratory exercise testers were blinded to the participant’s intervention arm at all times. Exercise trainers and study staff asked about adverse events at every contact. Severity and relationship of adverse events to intervention was determined by an un-blinded clinician investigator. An independent safety committee reviewed adverse events quarterly.

### Outcome measures

Our primary outcome measures of interest included co-primary composite scores of memory and executive function domains. These outcome measures represent our single primary outcome upon which the study was powered. Our secondary aim focused on testing the effect of exercise on function (Disability Assessment of Dementia [DAD]) and depressive symptoms (Cornell Scale for Depression). We did not adjust for multiple testing as this study was designed and funded as a pilot study to generate data for designing more definitive trial.

A comprehensive cognitive test battery was given at baseline and repeated at Week 13 and Week 26, employing validated, alternate versions of tests every other visit. Based on recent recommendations,[30] our primary outcome analyses used standardized composite scores of memory and executive function (S1 Table).[31] Planned tests for the memory composite score were Logical Memory (Immediate and Delayed), Free and Cued Selective Reminding Test (sum of free recall), and Boston Naming Test. Based upon recent work noting uncertain psychometric properties [32] and to be consistent with existing composite factors,[33] we removed Boston Naming Test from our composite memory score. The Executive Function composite score was comprised of Digit Span (Forward and Backward), Category Fluency, D-KEFS Confirmed Correct and Free Card Sorting, Letter Number Sequencing and Stroop Color-Word Interference.

We created composite cognitive scores by averaging standardized scores within each domain. Raw scores were standardized to the mean and standard deviation of healthy older adult baseline scores from a companion study (M = 0, SD = 1).[34] The healthy older adult sample was recruited from the same geographic distribution during the same timeframe and using similar inclusion and exclusion criteria, making it an appropriate group with confirmed normal cognition on which to standardize performance as has been done previously.[33] On rare occasions (see S2 Table), participants were unable to complete a test due to the severity of their cognitive impairment. In these cases the score representing the lowest performance was given. When participants withdrew from the study, tests were marked as missing.

Our outcome measure of depression was the Cornell Scale for Depression in Dementia as rated by the caregiver.[35] Our outcome measure of functional ability was the Disability Assessment for Dementia, which asks the caregiver to assess independence in activities of daily living in the previous two weeks.[36]

We assessed cardiorespiratory fitness at baseline and Week 26 as the highest oxygen consumption attained (peak VO_2_) during cardiorespiratory exercise testing on a treadmill to maximal capacity or volitional termination.[37] Dual x-ray absorptiometry was performed (Prodigy, GE Healthcare, Milwaukee, WI) to allow for normalization of peak VO_2_ to lean mass.[11, 38] We normalized to lean mass because muscle accounts for the majority of oxygen uptake during exercise.[11, 39] The 6-minute walk test was performed as a secondary measure of functional fitness in a quiet, 100’ hallway.[40]

MRI was performed at baseline and following the intervention in a Siemens 3.0 Tesla scanner. We obtained a high-resolution T1 weighted image (MP-RAGE; 1x1x1mm voxels; TR = 2500ms, TE = 4.38ms, TI = 1100ms, FOV 256mmx256mm with 18% oversample, 1mm slice thickness, 8 degree flip angle) for detailed anatomical assessment. We used the Freesurfer image analysis suite (http://surfer.nmr.mgh.harvard.edu/) for volumetric segmentation optimized for longitudinal data,[41] extracting hippocampal and total gray matter volume as exploratory measures of brain health.

### Intervention

Participants were asked not to alter current physical activities other than those prescribed by the study team. The AEx group began the intervention with a weekly goal of 60min in Week 1 and increased their weekly exercise duration by approximately 21min per week until they achieved the current public health recommended target duration of 150min per week, distributed over 3–5 sessions. Target heart rate (HR) zones were gradually increased from 40–55% to 60–75% of HR reserve based on resting and peak HR during cardiorespiratory fitness testing. HR was monitored at the YMCA by conventions chest worn sensor (F4 or FT4, Polar Electro, Inc. Lake Success, NY). Total exercise duration and a rating of perceived exertion (Borg 6–20) were gathered during each session. Exercise trainers supervised all exercise sessions during Weeks 1–6 and gradually reduced supervised sessions to 1 per week based on perceived ability to be safe and independent and in consultation with the participant’s study partner and study staff.

The ST group performed a series of non-aerobic exercises that rotated weekly (core strengthening, resistance bands, modified tai chi, modified yoga). As in several previous studies [42–46] we chose an active control intervention (ST) to account for potential effects of social engagement and physical activity.[47] Participants in the ST group wore HR monitors and were asked to keep their HR below 100 beats per minute. Exercise trainers helped participants adjust exercise intensity to reduce HR as necessary. Similar to the AEx group, trainers supervised all exercise sessions during Weeks 1–6 and gradually reduced supervised sessions to 1 per week based on perceived ability to be safe and independent and in consultation with the participant’s study partner and study staff.

To standardize implementation of the intervention protocols a training manual was developed for exercise trainers. Study staff performed in-person training of the exercise trainers. This training was reinforced at the time of enrollment of new participants at which time the exercise trainer, study coordinator, and participant met at the YMCA to review the intervention. Study staff also performed bi-weekly visits to YMCA facilities to monitor fidelity to the intervention protocols. This training method has been used in a previous study and shown to rigorously control intervention.^25^

### Analysis

We performed a linear mixed-effects analysis of the effect of treatment arm on our primary outcomes using R[48] and the lme4 package to allow for missing data due to withdrawal.[49] Participants were included in analyses regardless of protocol adherence. We entered Treatment Arm (AEx, ST), Timepoint (Baseline, Week 13, Week 26) and Education into the model as fixed effects and included random intercepts for participants. P-values were obtained by likelihood ratio tests of the full model including the interaction of Timepoint and Treatment Arm against the model without the interaction. We followed a similar procedure for secondary measures (e.g. peak VO_2_, brain volumes) with only Baseline and Week 26 timepoints. Analyses were conducted with α = 0.05 to protect against Type I error. To estimate the effect sizes of our outcomes, we calculated estimated difference between groups at Week 26, adjusted for education.

To explore the hypothesis that any benefits would be proportional to cardiorespiratory fitness gain we also performed a multi-step, hierarchical linear regression of our primary and secondary outcomes against change in peak VO_2_ after correcting for age, sex, education.

## Results

### Participants

**Fig 1:**
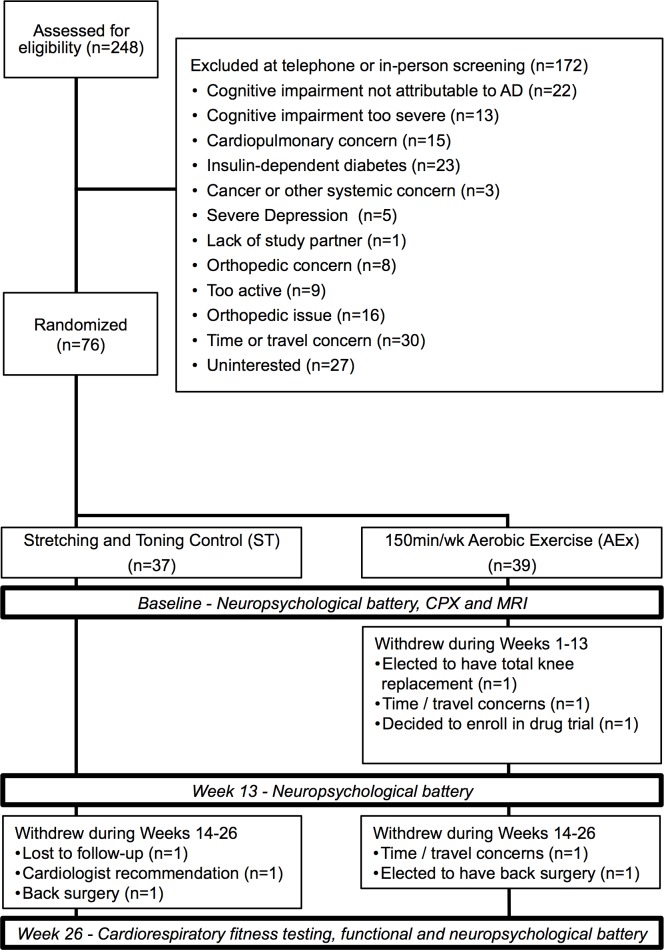
Study enrollment flow. CPX = cardiopulmonary exercise test, MRI = magnetic resonance imagery, AEx = Aerobic exercise condition, ST = stretching and toning control condition, AD = Alzheimer’s disease.

A total of 248 individuals were assessed for study eligibility. The flow of participants through screening to enrollment is shown in Fig 1. Participants (n = 76) were randomized to either the ST (n = 37) or AEx (n = 39) intervention groups. A total of 68 participants (89%: ST n = 34, AEx n = 34) completed the study. Demographic and baseline characteristics are given in Table 1. Participants in the two groups did not significantly differ in these measures.

**Table 1:** Participant Demographics.

	Stretching and Toning (n = 37)	Aerobic Exercise (n = 39)
Age (y)	71.4 (8.4]	74.4 (6.7)
Sex (#, % male)	16 (43.2%)	21 (56.8%)
Education (y)	16.1 (3.1)	15.5 (3.3)
ApoE ε4 carriers (#, %)	26 (70.3%)	18 (46.2%)
CDR Sum of Boxes	3.55 (1.6)	3.54 (1.99)
MMSE (units)	25.0 (3.2)	25.8 (3.3)
Lean Mass (kg)	48.9 (10.8)	48.0 (10.4)
Body Weight (kg)	79.2 (16.6)	78.1 (14.4)
Peak VO_2_ (mL·kg lean mass^-1^·min^-1^)	33.7 (8.0)	33.6 (5.5)

Mean (standard deviation) unless otherwise noted.

### Adherence to exercise protocol

The ST group averaged 79.9% (SD 20%) of the prescribed exercise dose as measured by total prescribed minutes of exercise and the AEx group completed 85% (SD 35%) of the prescribed exercise dose.

### Outcomes of interest

There was no apparent effect of intervention on primary outcome measures of Memory and Executive Function or in depressive symptoms (Table 2). AEx was associated with a modest gain in functional ability (Disability Assessment for Dementia) compared to individuals in the ST group (X^2^ = 8.2, p = 0.02). Estimated primary outcome effects of the difference between groups at Week 26, adjusted for education can be found in S3 Table.

**Table 2:** Primary Outcome Measures.

	Timepoint	Stretching and Toning Control	Aerobic Exercise	Arm by Timepoint Interaction
Memory Composite	Baseline	-2.8 (1.4)	-2.5 (1.4)	X^2^ = 0.82 (2) p = 0.66
	Week 13	-2.8 (1.5)	-2.3 (1.5)	
	Week 26	-2.7 (1.7)	-2.3 (1.7)	
Executive Function Composite	Baseline	-1.34 (0.85)	-1.12 (0.82)	X^2^ = 2.6(2), p = 0.27
	Week 13	-1.25 (0.94)	-1.09 (0.86)	
	Week 26	-1.33 (0.97)	-1.20 (0.90)	
Disability Assessment for Dementia	Baseline	91.2 (8.0)	88.0 (12.3)	X^2^ = 8.2(2), **p = 0.02**
	Week 13	89.5 (12.8)	89.8 (12.5)	
	Week 26	86.7 (13.3)	89.5 (13.7)	
Cornell Scale for Depression in Dementia	Baseline	7.4 (3.8)	8.6 (5.1)	X^2^ = 1.3(2), p = 0.51
	Week 13	8.1 (4.4)	8.4 (4.6)	
	Week 26	7.8 (4.4)	7.8 (5.2)	

Mean (standard deviation) unless otherwise noted.

### Secondary outcomes

We next assessed intervention effects on secondary outcome measures of cardiorespiratory fitness (peak VO_2_ and the 6-minute walk test) and brain structure (bilateral hippocampal volume and total gray matter). AEx was associated with increased performance on the 6-minute walk compared to ST (Timepoint by Treatment Arm interaction, X^2^ = 0.003) although AEx was not associated with benefits on other secondary measures (Table 3). Estimated secondary outcome effects of the difference between groups at Week 26, adjusted for education, can be found in S4 Table.

**Table 3:** Baseline and change at Week 26 secondary outcome measures.

Outcome measure	Timepoint	ST	AEx	Arm by Timepoint Interaction
Peak Oxygen Consumption (mL·kg lean mass^-1^·min^-1^)	Baseline	33.7(8.0)	33.6 (5.5)	X^2^ = 0.9, p = 0.35
	Δ	0.01 (3.0)	0.96 (4.4)	
6 minute walk (yds)	Baseline	473.6 (90.7)	465.9 (57.5)	X^2^ = 9.1, **p = 0.003**
	Δ	-33.4 (69.1)	18.7 (65.9)	
Bilateral Hippocampus (cm^3^)	Baseline	6.2 (1.3)	6.1 (1.3)	X^2^ = 1.0, p = 0.31
	Δ	-0.10 (0.24)	-0.05 (0.15)	
Total Gray Matter Volume (cm^3^)	Baseline	564 (64)	574 (54)	X^2^ = 1.3, p = 0.25
	Δ	-1.95 (15)	-6.18 (13)	

**Fig 2:**
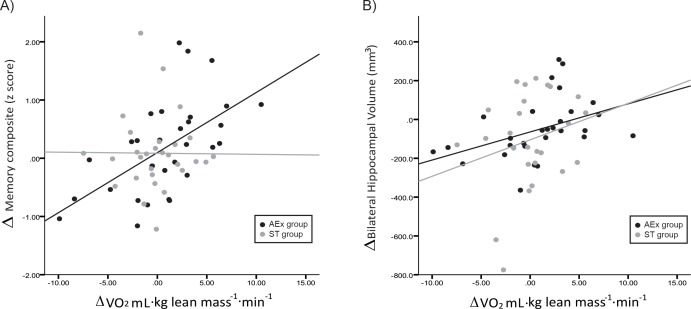
Relationship of change in peak VO_2_ with change in (A) memory composite score and (B) bilateral hippocampal volume. Blue data points represent the aerobic exercise group. Green data points represent the stretching and toning control group.

To explore possible fitness-specific effects, we also examined the relationship of change in cardiorespiratory fitness (peak VO_2_) with changes observed in outcome measures in the overall group (as both treatment arms were active interventions) and within intervention groups (Table 4 and Fig 2). For the overall group, change in peak VO_2_ was associated with change in the memory composite score (p = 0.003) and change in bilateral hippocampal volume (p = 0.03, Fig 2). Within group analyses demonstrated that cardiorespiratory fitness was primarily related to changes in memory performance and hippocampal volume in the AEx group (Table 4).

**Table 4:** Relationship of Change in Cardiorespiratory Fitness (VO_2_ peak) with Change in Primary and Secondary Outcome Measures.

	Overall	AEx Group	ST Group
Memory Composite	0.07 [0.02 0.12]**	0.10 [0.04 0.17]**	0.05 [-0.05 0.15]
Executive Function Composite	-0.02 [-0.05 0.01]	-0.02 [-0.06 0.02]	-0.0 [-0.06 0.05]
Disability Assessment for Dementia	-0.12 [-0.70 0.46]	-0.19 [-0.91 0.53]	-0.17 [-1.35 1.01]
Cornell Scale for Depression in Dementia	0.11 [-0.16 0.37]	0.12 [-0.18 0.42]	0.25 [-0.30 0.79]
Hippocampal Volume (cm^3^)	0.020 [0.001 0.029]*	0.014 [0.001 0.027]*	0.014 [-0.022 0.052]
Gray Matter Volume (cm^3^)	0.404 [-0.592 1.401]	0.318 [-0.800 1.436]	1.534 [-0.697 3.765]
6-minute walk (yds)	-2.28 [-7.61 3.04]	1.28 [-4.09 6.64]	-9.77 [-19.5–0.03]*

Unstandardized coefficients of the peak VO_2_ change term [95% confidence interval] are presented after controlling for age, sex and education. Lean mass was lost for 1 individual due to equipment malfunction; only 57 people completed imaging at both timepoints. p<0.05*, p<0.01**.

### Adverse events

There were 25 adverse events possibly or definitely related to the intervention or cardiorespiratory exercise testing: 7 mild, 2 moderate and 1 severe in the ST group and 14 mild and 1 of moderate severity in the AEx group. Common mild adverse events possibly or probably related to the intervention included low back, hip, knee or foot pain. Moderate severity adverse events included lower extremity pain (n = 4), heart rhythm abnormalities (n = 3), and chest pain (n = 1). The severe event was back pain related to spinal stenosis possibly exacerbated by exercise.

## Discussion

This pilot, randomized, controlled trial provides preliminary evidence that 6-months of AEx benefits functional ability in early-stage AD compared to a ST control intervention. Furthermore, we found evidence that improvements in cardiorespiratory fitness were related to benefits in memory performance and brain volume change. These data fit with a growing body of evidence that enhancing cardiorespiratory fitness through exercise may be important in attaining maximal brain benefits of exercise.[18, 34, 43, 50, 51]

Our primary finding is that 26 weeks of AEx was associated with increased functional ability compared to the ST control. Our measure of functional ability was the Disability Assessment for Dementia, a caregiver-based assessment of activities of daily living, that predicts earlier time to institutionalization.[52] Individuals with mild to moderate AD typically decline approximately 1 point per month on this scale (100 equates to full functional ability).[36] We found that the AEx group increased 1.5 points while the ST group decreased 4.5 points over the course of the intervention suggesting a meaningful effect on sustained independence. This also extends prior findings suggesting that exercise promotes function in AD.[21, 53]

We also found that exercise-related change in the gold standard measure of cardiorespiratory fitness (peak VO_2_) was related to change in both memory performance and bilateral hippocampal volume. This observation supports the concept, reported widely in animal data, that exercise may attenuate AD-related brain and cognitive decline although CR fitness gains may be necessary to achieve these benefits These findings thus support the cardiorespiratory fitness hypothesis,[42] which posits that improved fitness as a result of aerobic exercise is essential and causally related to attaining exercise-related cognitive benefits. The cardiorespiratory fitness hypothesis is also supported by human studies that suggest cardiorespiratory fitness gains may be important in mediating physiological benefits to brain health.[18, 34] Gains in cardiorespiratory fitness may reflect complex systemic changes necessary to impact brain physiology or reflect an individual’s ability to attain sufficient levels of exercise necessary to impact brain structure and function.

Surprisingly, we did not observe significant group differences in exercise-related gains in cardiorespiratory fitness (peak VO_2_) for the AEx group (3% gain) compared to the ST group (0.03%). The modest 3% gain in cardiorespiratory fitness for the AEx group is lower than what we achieve in cognitively normal older adults following a similar protocol [34] despite good compliance with the exercise protocol and an increase in our secondary measure of functional fitness, the 6-minute walk test. This lack of a robust peak VO_2_ response suggests that individuals with early AD may have a limited, or more variable, physiologic response to aerobic exercise than cognitively normal individuals. We have previously reported that individuals with AD exhibit lower cross-sectional peak VO_2_ and significantly greater longitudinal peak VO_2_ decline than cognitively normal older adults.[12] This suggests that there may be inherent physiological differences, beyond behavioral issues, that may limit cardiorespiratory fitness responses.

The primary limitation for this pilot study is a relatively small sample size that limits our power to detect significant group effects. The exercise interventions were delivered in the community, enhancing generalizability but possibly introducing variability in execution, though we have previously demonstrated that our community-based methods can deliver a rigorously-controlled intervention of various exercise doses producing linearly increasing responses to cardiorespiratory fitness.[34] Our findings of a relationship of change in cardiorespiratory fitness with memory change and hippocampal atrophy are suggestive but do not prove cause and effect. For instance, it remains unclear whether improvement in cardiorespiratory fitness drives memory improvement or whether a decline in memory (or more severely progressive dementia) influences measured cardiorespiratory fitness as indexed by peak VO_2._ Reverse causation cannot be ruled out as an explanation for these secondary findings, although these relationships remained significant even when controlling for baseline MMSE or baseline CDR (as an index of baseline disease severity). Finally, it is important to note that we made no correction for multiple tests, raising the potential for false positives. While multiple outcomes are not ideal for a clinical trial, we felt it important to explore in this pilot study the various aspects of function that have previously been shown to benefit from aerobic exercise.

In conclusion, findings from this pilot randomized controlled trial were consistent with previous work showing aerobic exercise benefits functional ability in individuals with early-stage AD. Further, we found indirect evidence that exercise-related increases in cardiorespiratory fitness may be important to improving memory performance and reducing hippocampal atrophy. These effects should be explored in a definitive trial of aerobic exercise for individuals with early-stage AD. We have provided effect size estimates and confidence intervals to inform these future studies.

## Supporting information

S1 Checklist CONSORT checklist. (PDF)

S1 Table Raw Cognitive Test Scores. (DOCX)

S2 Table Missing Test Scores Due to Unable-to-Complete. (DOCX)

S3 Table Estimated effect of the difference in baseline to week 26 change in the AEx group versus baseline to week 26 change in the ST group, adjusted for education. (DOCX)

S4 Table Estimated effect of the difference between groups at Week 26, adjusted for education. (DOCX)

S1 Protocol Study protocol. (PDF)
