# Supplementary material for: Aerobic exercise for Alzheimer's disease: A randomized controlled pilot trial
Source: PLoS One. 2017 Feb 10;12(2):e0170547. doi: 10.1371/journal.pone.0170547 (PMC5302785; doi:10.1371/journal.pone.0170547)
Supplement: S1 Table — (DOCX) [file pone.0170547.s002.docx]

S1 Table – Raw Cognitive Test Scores

| **Neuropsychometric Test Outcomes**  **(Raw Scores)** | | **ST Intervention** | | | **AEx Intervention** | | |
| --- | --- | --- | --- | --- | --- | --- | --- |
|  |  | **T1 (n=37)** | **T2 (n=37)** | **T3 (n=34)** | **T1 (n =39)** | **T2 (n=35)** | **T3 (n=34)** |
| Memory Composite | Logical Memory (Immediate) | 6.6 [4.4] | 7.0 [5.0] | 7.0 [5.9] | 7.2 [4.2] | 8.0 [4.5] | 7.6 [4.9] |
|  | Logical Memory (Delayed) | 3.3 [4.5] | 3.6 [5.0] | 4.1 [5.9] | 4.6 [4.4] | 5.3 [5.1] | 5.4 [5.3] |
|  | Total Free Recall | 12.8 [8.8] | 11.6 [8.9] | 11.9 [9.8] | 14.9 [9.8] | 15.2 [9.9] | 15.4 [11.0] |
| Executive Function Composite | Digit Span Forward | 7.1 [1.7] | 7.4 [2.0] | 7.3 [2.0] | 7.5 [2.0] | 7.5 [1.9] | 7.3 [1.8] |
|  | Digit Span Backward | 5.0 [1.8] | 5.1 [2.2] | 4.8 [2.1] | 4.9 [2.1] | 5.2 [2.1] | 4.8 [1.9] |
|  | Category Fluency (Animals) | 14.0 [4.9] | 13.3 [5.4] | 13.9 [5.3] | 14.9 [5.8] | 14.5 [4.5] | 14.4 [5.7] |
|  | Category Fluency (Vegetables) | 8.8 [3.7] | 8.8 [4.3] | 8.2 [4.2] | 9.3 [3.1] | 9.4 [3.9] | 9.4 [4.5] |
|  | DKEFS Free Sort 1 | 10.0 [7.0] | 10.6 [6.7] | 10.4 [7.7] | 10.3 [6.0] | 11.0 [6.5] | 11.5 [7.0] |
|  | DKEFS Free Sort 2 | 8.7 [6.3] | 9.4 [8.3] | 9.2 [7.5] | 11.5 [6.6] | 11.0 [5.8] | 11.4 [6.7] |
|  | DKEFS Confirmed Correct Perceptual Sorts (Deck 1) | 1.5 [1.4] | 1.6 [1.4] | 1.7 [1.4] | 1.6 [1.2] | 1.9 [1.4] | 2.0 [1.6] |
|  | DKEFS Confirmed Correct Perceptual Sorts (Deck 2) | 1.4 [1.3] | 1.4 [1.5] | 1.4 [1.5] | 1.9 [1.5] | 1.9 [1.2] | 1.9 [1.3] |
|  | Letter Number Sequencing | 5.2 [3.5] | 5.3 [3.4] | 5.4 [3.4] | 6.5 [3.4] | 6.1 [3.4] | 6.3 [3.6] |
|  | Stroop | 23.2 [12.4] | 25.2 [12.5] | 23.9 [13.7] | 23.3 [13.4] | 25.7 [13.9] | 23.6 [14.4] |
