# Supplementary material for: Aerobic exercise for Alzheimer's disease: A randomized controlled pilot trial
Source: PLoS One. 2017 Feb 10;12(2):e0170547. doi: 10.1371/journal.pone.0170547 (PMC5302785; doi:10.1371/journal.pone.0170547)
Supplement: S2 Table — (DOCX) [file pone.0170547.s003.docx]

S2 Table – Missing Cognitive Test Scores Due to Unable-to-Complete

| **Neuropsychometric Test Outcomes**  **Number Missing but not Withdrawn** | | **ST Intervention** | | | **AEx Intervention** | | |
| --- | --- | --- | --- | --- | --- | --- | --- |
|  |  | **T1** | **T2** | **T3** | **T1** | **T2** | **T3** |
| Memory Composite | Logical Memory (Immediate) | 0 | 0 | 0 | 0 | 0 | 0 |
|  | Logical Memory (Delayed) | 0 | 0 | 0 | 0 | 0 | 0 |
|  | Total Free Recall | 0 | 0 | 1 | 0 | 0 | 0 |
| Executive Function Composite | Digit Span Forward | 0 | 0 | 0 | 0 | 0 | 0 |
|  | Digit Span Backward | 0 | 0 | 0 | 0 | 1 | 0 |
|  | Category Fluency (Animals) | 0 | 0 | 0 | 0 | 0 | 0 |
|  | Category Fluency (Vegetables) | 0 | 0 | 0 | 0 | 0 | 0 |
|  | DKEFS Free Sort 1 | 0 | 0 | 0 | 0 | 0 | 0 |
|  | DKEFS Free Sort 2 | 0 | 0 | 0 | 0 | 0 | 0 |
|  | DKEFS Confirmed Correct Perceptual Sorts (Deck 1) | 0 | 0 | 0 | 0 | 0 | 0 |
|  | DKEFS Confirmed Correct Perceptual Sorts (Deck 2) | 0 | 0 | 0 | 0 | 0 | 0 |
|  | Letter Number Sequencing | 0 | 6 | 2 | 0 | 3 | 2 |
|  | Stroop | 0 | 1 | 2 | 0 | 2 | 1 |
| Depression | Cornell Scale for Depression in Dementia | 1 | 0 | 0 | 0 | 0 | 0 |
| Disability | Disability Assessment for Dementia | 0 | 0 | 0 | 0 | 0 | 0 |
