# Supplementary material for: Aerobic exercise for Alzheimer's disease: A randomized controlled pilot trial
Source: PLoS One. 2017 Feb 10;12(2):e0170547. doi: 10.1371/journal.pone.0170547 (PMC5302785; doi:10.1371/journal.pone.0170547)
Supplement: S3 Table — (DOCX) [file pone.0170547.s004.docx]

| **S3 Table.** Estimated effect of the difference between AEx and ST groups at Week 26, adjusted for education. | |
| --- | --- |
|  | Estimated Effect [95% Confidence Interval] |
| Memory Composite | 0.08 [-0.21 0.38] |
| Executive Function Composite | -0.13 [-0.33 0.06] |
| Disability Assessment for Dementia | 5.27 [1.7 8.84] |
| Cornell Scale for Depression in Dementia | -0.98 [-2.65 0.69] |
