# Supplementary material for: Aerobic exercise for Alzheimer's disease: A randomized controlled pilot trial
Source: PLoS One. 2017 Feb 10;12(2):e0170547. doi: 10.1371/journal.pone.0170547 (PMC5302785; doi:10.1371/journal.pone.0170547)
Supplement: S4 Table — (DOCX) [file pone.0170547.s005.docx]

**S4 Table.** Estimated effect of the difference between groups at Week 26, adjusted for education.

| Secondary Outcome | Estimated Effect [95% Confidence Interval] |
| --- | --- |
| Peak Oxygen Consumption  (mL·kg lean mass^-1^·min^-1^) | 0.88 [-0.95 2.71] |
| 6 minute walk (yds) | 25.91 [9.65 42.16] |
| Bilateral Hippocampus (cm^3^) | 0.05 [-0.05 0.15] |
| Total Gray Matter Volume (cm^3^) | -4.23 [-11.37 2.92] |
