# Supplementary material for: Aerobic exercise for Alzheimer's disease: A randomized controlled pilot trial
Source: PLoS One. 2017 Feb 10;12(2):e0170547. doi: 10.1371/journal.pone.0170547 (PMC5302785; doi:10.1371/journal.pone.0170547)
Supplement: S1 Protocol — (PDF) [file pone.0170547.s006.pdf]

Contemporary Clinical Trials 33 (2012) 1105–1116

Contents lists available at SciVerse ScienceDirect

Contemporary Clinical Trials

journal homepage: www.elsevier.com/locate/conclintrial

A community-based approach to trials of aerobic exercise in aging and Alzheimer's disease

Eric D. Vidoni [a,g], Angela Van Sciver [a,g], David K. Johnson [b,g], Jinghua He [c], Robyn Honea [a], Brian Haines [d], Jami Goodwin [a,g], M. Pat Laubinger [a], Heather S. Anderson [a,g], Patricia M. Kluding [e], Joseph E. Donnelly [f], Sandra A. Billinger [e], Jeffrey M. Burns [a,g,*]

[a] Dept. of Neurology, University of Kansas Medical Center, Kansas City, KS, USA
[b] Dept. of Psychology, University of Kansas, Lawrence, KS, USA
[c] Dept. of Biostatistics, University of Kansas Medical Center, Kansas City, KS, USA
[d] YMCA of Greater Kansas City Inc., Kansas City, MO, USA
[e] Dept. of Physical Therapy and Rehabilitation Science, University of Kansas Medical Center, Kansas City, KS, USA
[f] Cardiovascular Research Institute, University of Kansas Medical Center, Kansas City, KS, USA
[g] University of Kansas Alzheimer's Disease Center, Fairway, KS, USA

ARTICLE INFO

Article history:
Received 24 March 2012
Received in revised form 1 August 2012
Accepted 3 August 2012
Available online 7 August 2012

Keywords:
Randomized controlled trial
Dementia
Public–private partnership

ABSTRACT

The benefits of exercise for aging have received considerable attention in both the popular and academic press. The putative benefits of exercise for maximizing cognitive function and supporting brain health have great potential for combating Alzheimer's disease (AD). Aerobic exercise offers a low-cost, low-risk intervention that is widely available and may have disease modifying effects. Demonstrating that aerobic exercise alters the AD process would have enormous public health implications. The purpose of this paper is to report the protocol of a current, community-based pilot study of aerobic exercise for AD to guide future investigation. This manuscript provides 1) an overview of possible benefits of exercise in those with dementia, 2) a rationale and recommendations for implementation of a community-based approach, 3) recommendation for implementation of similar study protocols, and 4) unique challenges in conducting an exercise trial in AD.

© 2012 Elsevier Inc. All rights reserved.

1. Introduction

Americans are living longer than ever before. Six percent of the population is 75 years or older and this age group is expected to increase by 80% in the next decade [1,2]. Aging brings an increased incidence of cognitive and physical decline, especially in those in the eighth decade and beyond [3]. One in 8 individuals over 65 is now living with Alzheimer's disease (AD), [4] and the annual treatment costs of AD are estimated at $183 billion in the US. Spending on dementia care is expected to increase 600% over the next 40 years [4]. Delaying the onset or slowing the progression of AD would significantly reduce annual health care costs in the US [5]. However, there are currently no established and accepted disease modifying or preventive treatments for AD.

A wealth of animal research data suggests that exercise positively impacts brain health. Increased physical activity may have a trophic effect on the brain, particularly the hippocampus. For instance, exercise increases brain-derived neurotrophic factor (BDNF) [6] and other important neurochemicals [7] supporting brain growth and survival. Exercise appears to stimulate neurogenesis [8], enhance neuronal survival [9], increase resistance to brain insults [10,11] and increase synaptic

Abbreviations: ADEPT, Alzheimer's Disease Exercise Program Trial;CDR, Clinical Dementia Rating;MCI, mild cognitive impairment;AD, Alzheimer's disease;the Y, YMCA of Greater Kansas City;THR, target heart rate;KU ADC, University of Kansas Alzheimer's Disease Center;CPT, certified personal trainer;CTSU, Clinical and Translational Science Unit

* Corresponding author at: KU Alzheimer's Disease Center, 4350 Shawnee Mission Parkway, MS 6002, Fairway, KS 66205, USA. Tel.: +1 913 588 0555; fax: +1 913 945 5035.
E-mail address: jburns2@kumc.edu (J.M. Burns).

1551-7144/$ – see front matter © 2012 Elsevier Inc. All rights reserved.
http://dx.doi.org/10.1016/j.cct.2012.08.002

plasticity [12]. Exercise promotes brain vascularization [13,14], mobilizes gene expression profiles predicted to benefit brain plasticity [15], and maintains cognitive function [16]. Additionally, exercise effects on the brain may reduce vascular risk factors (heart disease [17], atherosclerosis [18], stroke [19], and diabetes [20–24]) that are believed to place an individual at risk for dementia, vascular dementia, and AD [25].

Further, there is limited but compelling animal data suggesting that exercise may have disease-modifying benefits in AD. For instance, increased physical activity in mouse models of AD reduces neuropathological burden [26] and may promote hippocampal neurogenesis [27]. The reduction of β-amyloid in the exercising mice was evident in both cortical and hippocampal regions of the brain suggesting that voluntary exercise may mediate the amyloid cascade in favor of reduced production of β-amyloid [26]. Despite the evidence that aerobic exercise may be disease modifying in animals, there is a paucity of rigorous trials of aerobic exercise effects on cognitive function in the earliest stages of AD in humans [28]. The lack of well-designed, randomized controlled trials (RCT) that have investigated exercise effects on cognition has led to scientific position statements [29] and prominent public media editorials [30] on the insufficiency of evidence for addressing cognitive decline. Studies of exercise in dementia are significantly limited by poorly-defined samples and insufficient outcome measures and exercise regimens. Many of these studies include poorly characterized participants or use insensitive cognitive and physical outcome measures. For example, one meta-analysis of older studies suggested physical exercise can benefit physical and cognitive performance [31] but the results are derived from a mix of exercise modalities and dementia states [32–53]. Often these mixed studies include institutionalized elderly patients in nursing homes or psychiatric hospitals with poorly characterized cognitive impairment. Few focus on diagnosing AD [40,54] and fewer examine individuals with early stage AD [55]. Exercise interventions are variable and usually involve resistance (sit-to-stand exercises, strength training, isometric exercises in sitting positions) [41,42,45,47,56,57] and endurance training [53,58,59]. Outcome variables vary widely and include mobility and balance, strength, reaction time, and functional measures [31]. Endurance measures are generally the distance walked over a set time (i.e., modified 6-minute walk). To our knowledge, few studies include standard measures of exercise adaptation such as insulin sensitivity, body composition, lipids, and VO_2 peak in people with well-characterized early stage dementia.

There is promising evidence that aerobic exercise benefits brain health and cognitive function in AD. Cross-sectional evidence from our center has found that peak oxygen consumption (VO_2 peak, a consensually valid measure of aerobic fitness and standard outcome measure of aerobic exercise interventions) in early-stage AD is associated with whole brain volume measures, with higher VO_2 peak associated with less brain atrophy and slower dementia progression [60–62]. In one recent randomized controlled trial (RCT) for individuals with MCI, aerobic exercise improved executive cognitive function, especially in women [55]. Importantly, this study demonstrated that a similar, community-based approach was feasible and has the potential to modify cognitive measures. Exercise may also improve mood and functional capacity [40]. Ongoing RCTs of home-based programs will shed further light on the role of exercise for managing AD, but there is a need for more research among community-dwelling adults in the earliest stages of AD.

While it is common for clinicians to recommend a physically active lifestyle to those with AD, the benefits of exercise in AD are not well-defined and evidence to develop guidelines for the prescription of exercise in AD is lacking [29,63]. Though physical activity typically declines with cognitive impairment [64], a meta-analysis demonstrated physical activity interventions to be successful for improving physical and functional performance as well as cognition [31] suggesting that improving exercise habits is feasible for this population. Although there is consensus that current recommendations (~30 min of exercise most days of the week) provide general health benefits to older adults [65], there remains a need for well-designed RCTs to test the long-term benefits of exercise for preventing cognitive and functional decline. Rigorous RCTs are challenging for a number of reasons including but not limited to their expense, need for control interventions, and recruitment of a cohort of sufficient size. These challenges are magnified by the unique needs of those with AD and their caregivers.

Aerobic exercise offers a low-cost, low-risk intervention that is widely-available and may have disease modifying effects. Demonstrating aerobic exercise alters the AD process would have enormous public health implications. The protocol outlined in this report builds on the strengths from prior programs [40,55] and capitalizes on existing and well recognized community resources. Additionally, it uses publically endorsed exercise recommendations, while providing information on cognitive, functional and physiologic measures that may be sensitive to aerobic exercise training in individuals in the earliest stages of AD.

2. Methods

The University of Kansas Alzheimer's Disease Center (KU ADC) is currently supporting a trial of aerobic exercise for cognition; the Alzheimer's Disease Exercise Program Trial (ADEPT) for adults over 55 in the earliest stages of AD. Personalized aerobic exercise programs are carried out at the Young Men's Christian Association of Greater Kansas City (the Y) locations.

2.1. Study design and specific aims

ADEPT is a 26 week RCT of aerobic exercise vs. non-aerobic activities in individuals 55 years of age and older in the earliest stages of AD. Participants are randomized to a control arm (non-aerobic activities) or treatment arm of 150 min per week of aerobic exercise, typically treadmill walking. All measures are assessed at baseline. Cognitive testing is repeated at Week 13 and all measures are repeated after Week 26.

This study is designed to generate efficacy data to inform a larger multi-site trial on our theories regarding the potential positive benefits of aerobic fitness with respect to cognition (Aim 1), and physical functioning and mental well being (Aim 2). It also seeks to explore potential disease-modifying benefits and potential mechanisms relating aerobic fitness with brain health. Aim 1 uses co-primary outcome

measures of memory and executive function data over 26 weeks in subjects participating in aerobic exercise vs. non-aerobic activities in individuals in the earliest clinical stages of MCI and AD. Aim 2 uses co-primary outcomes include the Disability Assessment in Dementia (daily function) and the Cornell Depression Scale (behavior) to quantify physical functioning and mental well being.

Two exploratory aims are designed (Aim 3) to refine neuroimaging biomarker measures to assess potential disease-modifying effects of exercise (global and hippocampal brain volume change over 26 weeks) and (Aim 4) to examine potential underlying systemic mechanisms relating fitness with brain health (insulin, insulin-like growth factor c-reactive protein, interleukin-6, and TNF-alpha).

2.2. Subject recruitment

The KU ADC has established an infrastructure for the identification, recruitment, and characterization of older adults both with and without dementia. Beginning in 2004, we developed a registry of individuals who have consented to be contacted regarding research studies. Other institutional databases of older adult research participants supplement our contact list of potential enrollees through mailings. In addition, the KU ADC and the Y have jointly designed a recruitment and marketing plan. Marketing materials (flyers, posters, postcards) are distributed at the Y and community locations, based on the population of older adults. KU ADC staff distributes study information in health fairs across the Kansas City community, conducting memory and physical function screens to emphasize the importance of physical activity and wellness. Often these are joint appearances with KU ADC and the Y staff to highlight the partnership in the community. Study staff also meets with medical residents and physicians to share information about the study. Family physicians, cardiologists, and neurologists are given pocket-sized laminated cards with basic study qualifications. These physicians are encouraged to share program information with qualified participants.

To date, print advertisements, clinic referrals and “word-of-mouth” have been the most effective recruitment tool, together accounting for 2/3 of referrals to our study. These tools are among the least expensive per participant yielded. Table 1 provides more detailed information on recruitment tool efficacy. All potential participants, whether referred through community events or their physician, are directed to a KU ADC recruitment coordinator for a brief telephone screening.

All potential participants must have a study partner with whom they are in regular contact. The study partner is asked to accompany the person to the initial examination and to serve as a collateral source for evaluations. Although the study partner is not required to accompany the participant to exercise sessions it is strongly encouraged for those who have limited independence. The study provides a household membership to the Y to allow for this participation.

2.3. Screening

Prior to enrollment and randomization, all participants undergo a standard evaluation in multiple visits at the KU ADC.

Table 1
ADEPT inclusion and exclusion criteria.

Inclusion criteria	Exclusion criteria
• Informed consent<br>• Age 55 years or older<br>• Underactive or sedentary based on the Telephone Assessment of Physical Activity [95].<br>• Community dwelling with a caregiver willing to accompany the participant to visits to the screening evaluation. The caregiver must visit with the subject more than five times a week.<br>• CDR 0.5 or 1<br>• Adequate visual and auditory abilities to perform all aspects of the cognitive and functional assessments<br>• Stable doses of medications for at least 30 days prior to screening.<br>• Likely to participate in all scheduled evaluations and complete the exercise program over 26 weeks	• Current clinically significant major psychiatric disorder (e.g., major depressive disorder) according to DSM-IV criteria or Geriatric Depression score of 5 or greater.<br>• Significant psychiatric symptoms (e.g., hallucinations) that could impair the completion of the study<br>• Current clinically-significant systemic illness likely to result in deterioration of the patient's condition or affect the patient's safety during the study<br>• History of clinically-evident stroke<br>• Clinically-significant infection within the last 30 days<br>• Myocardial infarction or symptoms of coronary artery disease (e.g., angina) in the last two-years.<br>• Uncontrolled hypertension within the last 6 months<br>• History of cancer within the last 5 years (except non-metastatic basal or squamous cell carcinoma)<br>• History of drug or alcohol abuse as defined by DSM-IV criteria within the last 2 years<br>• Insulin-dependent diabetes mellitus<br>• Significant pain or musculoskeletal disorder that would prohibit participation in an exercise program

Prior studies have used an extended “run-in” period with multiple visits to ensure commitment and availability [66]. The screening and baseline testing process requires 3–4 visits over a 30-day period and takes approximately 9 h to complete. All visits occur at the University of Kansas Clinical and Translational Science Unit (CTSU) or the Hoglund Brain Imaging Center, on the University of Kansas Medical Center campus. Appointments are combined when scheduling allows.

All participants receive a thorough clinical evaluation by a clinician through the KU ADC which lasts about 1.5 h. The clinical evaluation includes a Clinical Dementia Rating (CDR) [79,80], and semi-structured interviews with the participant and the study partner. During the evaluation the clinician reviews the clinical history, past medical history, and medications. The clinician also performs a physical and neurological examination and reviews inclusion and exclusion criteria (Table 2).

The clinical evaluation results are used to determine a presumptive clinical diagnosis which is reviewed and finalized at a consensus diagnostic conference attended by KU ADC clinicians and psychometricians using the NINDS-ADRDA criteria [67]. These methods have a high diagnostic accuracy for AD of 93% [68]. At the consensus conference, the examining clinician presents a brief clinical synopsis and CDR staging,

Table 2
Recruitment cost per referral.

Medium	Cost	Calls generated	Cost per call
Direct to home advertisement mailing (Valpak)	$3480	96	$36.25
Print advertisement	$1599	221	$7.24
Direct mail campaigns to existing research databases	$629	78	$8.07
Brochures in physician clinics and Y facilities	$308	15	$20.51

followed by a discussion of neuropsychological test results, and available clinical information from outside evaluations (i.e., MRI, lab tests). Per recently updated criteria [69], individuals classified with MCI are also assigned an etiologic diagnosis (e.g. AD, cerebrovascular disease, depression). Individuals classified as MCI who also demonstrate evidence of longitudinal decline and characteristic objective cognitive findings are assigned an etiologic diagnosis of probable AD.

If the individual meets inclusion criteria and has been assigned an etiologic diagnosis of AD, screening continues with physical function testing and a graded maximal exercise test which last approximately 2 h. Testing is performed using a Modified Bruce protocol in the presence of a medical monitor, exercise physiologist, nurse and spotter. Procedures for the exercise test have been previously reported [70] and are reliable in this population [71]. All participants are required to achieve a respiratory exchange ratio of at least 1.0 during the exercise test to be randomized [72]. Those not meeting this threshold are asked to return for an additional maximal exercise test. If it appears that orthopedic issues or participant concerns are limiting performance, the retest can be performed on a total body recumbent stepper [73]. If there are no orthopedic, cardiovascular or safety concerns raised during testing (e.g. abnormal EKG, poor safety awareness) the participant is enrolled in the study.

Participants who do not meet inclusion and exclusion criteria or are otherwise identified as inappropriate for the study are considered screen failures. For those enrolled, 2 additional visits are required: a 15 minute fasting blood draw performed by a CTSU nurse and an MRI visit that lasts approximately 2 h performed by study staff and a certified imaging technologist with experience in research protocols. Both visits are described in the Outcome measures section. Tests and measures acquired during the screening process are considered baseline measures for enrollees. Although this extensive testing battery may be burdensome to some participants, successful completion of all baseline measures is an excellent indicator of study completion and exercise adherence. In addition, our baseline screening procedures provide excellent characterization of study participants, including a detailed assessment of dementia and cognition, a limitation of previous studies.

2.4. Outcome measures

Cognitive testing is performed by a trained psychometrician at a separate visit lasting approximately 3 h. (see Table 3 for tests, descriptions, and anticipated effect sizes). The test battery includes standard measures of Verbal Memory, Executive Functioning (set maintenance and switching), Visuospatial Construction, and Reasoning. Verbal Memory and Executive Function factors serve as the primary outcomes measures for Aim 1. We use Confirmatory Factor Analysis (CFA) on baseline cognitive scores to create factor score weights which are then applied across the 3 times of measurement in this study. The Verbal Memory and Executive Function factors (primary outcomes of Aim 1) are previously validated in this neuropsychological battery [74,75]. Thus we use the CFA as a weighting schema to create more sensitive and specific summary scores (individual subtest loadings are not tested) shown to be excellent discriminators of healthy and pathological aging in both cross-sectional [74,76,77] and longitudinal [78] studies of Alzheimer's disease.

The study partner is asked to rate participants using the Quality of Life [79], Cornell Scale for Depression in Dementia [80], Disability Assessment for Dementia [81], Resource Utilization in Dementia — Lite [82], Alzheimer's Disease Cooperative Study Activities of Daily Living [83], and Epworth Sleepiness [84] scales. The Disability Assessment for Dementia and Cornell Scale for Depression in Dementia serve as primary outcome measures for Aim 2.

Overnight fasting blood glucose is tested and a blood sample banked for Exploratory Aim 2. The blood draw takes approximately 15 min and is often combined with another appointment such as the MRI or cognitive testing. When appointments are combined with a fasting lab draw, the participant is provided a voucher to eat after the fasting lab draw and prior to their next appointment.

The physical function and exercise test visit consists of a battery of performance and anthropometric measures. Waist and hip circumference, body weight and height and dual X-ray absorptiometry (DXA) are performed with the participant in a standard hospital gown with shoes removed. Then, the Physical Performance Test [85], Senior Fitness Test [86], and the graded maximal treadmill test. These measures serve as indices of training effect.

Finally, all participants undergo magnetic resonance imaging (MRI) of the brain in a Siemens 3.0 T MRI scanner which takes no more than 1 h. We obtain a high resolution T1 weighted (MPRAGE) image (MP-RAGE; $1\times1\times1$mm voxels; TR = 2500, TE = 4.38, TI = 1100, FOV 256×256 with 18% oversample, 1 mm slice thickness, flip angle 8°) for detailed anatomy with high gray-white matter contrast and a Fluid Attenuated Inversion Recovery (FLAIR) sequence for improved imaging of the periventricular white matter and discrimination of perivascular spaces from infarcts ($1\times1\times4$ mm voxels; flip angle = 180°; TI = 2500, TR = 10,000 s, TE = 81.0 ms). We use voxel based morphometry (VBM) analysis of the MRI as our imaging outcome to explore exercise-related effects on brain atrophy. VBM examines the whole brain and selected regions of interest in an unbiased way with high sensitivity to identify small changes in brain structure over time. Data analysis is performed using the VBM8 toolbox (http://dbm.neuro.uni-jena.de), an extension of the SPM8 algorithms (Wellcome Department of Cognitive Neurology, London, UK) running under MATLAB 7.3 (The MathWorks, Natick, MA, USA) on Linux. Every scan is checked for image artifacts and gross anatomical abnormalities. Subjects are excluded for movement artifact or any inhomogeneity that distorts brain matter.

Table 3
Cognitive test battery and reported effect sizes of exercise.

	Description	Effect size range
**Verbal Memory**		
Selective Reminding Test [96]	Picture list learning with structured category-cued reminding and uncued recall.	**0.16 to 0.59** [97,98]
Logical Memory [a,b] [99]	Prose recall of short narrative passages.	**0.02 to 1.44** [100–106]
Boston Naming Test[a] [107]	Naming of figural line drawings.	
Executive function		
Stroop Color-Word Test	Word reading and color naming requiring set maintenance and inhibition of irrelevant information.	**0.09 to 0.40** [97,98,102,108–110]
D-KEFS Card Sort	Card-sort requiring concept formation, set maintenance, and switching.	**0.08 to 1.61** [98,111,112]
Category Fluency[a] [113]	Spoken wordlist generation to a target category (Animals or Vegetables).	**0.17 to 1.16** [97,98,108,112,114,115]
Letter–Number Sequencing	Immediate memory for numbers and letter, requiring information to be maintained and manipulated in mind by self-ordered recall of like sets.	NA
Digit Span (Forward and Backward)[a] [99]	Immediate memory for numbers, requiring information to be maintained and manipulated in mind.	**0.05 to 3.50** [97,100,102–104,106,108,110,116–123]
**Visuospatial Construction**		
Digit Symbol[a] [124]	Test of psychomotor speed involving sustained attention and transcription of digit-symbol pairs.	**0.11 to 0.77** [98,101–104,106,108–110,112,114,115,117,118,125]
Trail Making Test[a] (Parts A and B) [126]	Line drawing between alternating numbers and letters, requiring visual search, attention, and mental flexibility.	**0.06 to 0.82** [102,106,108,109,112,114–117,121]
Block Design [124]	Visuospatial construction using blocks to match target models	NA
**Reasoning**		
Inductive Reasoning † [127]<br>Letter Series<br>Word Series<br>Number Series	Inference making based on principles derived from informative models containing letters, words, and numbers.	NA
Matrix Reasoning [124]	Inference making based on principles derived from informative models containing colored patterns.	**0.21 to 1.18** [105,111,112,121]
Picture Arrangement [124]	Card-sort requiring temporal sequencing and social reasoning.	**0.19** [123]
**Functional disability**		
Disability Assessment for Dementia [128]	Scale of need for assistance on basic and instrumental activities of daily living.	**0.4-0.8** [129]
Cornell Scale for Depression in Dementia [130]	Scale combining participant and informant ratings of mood and behavior.	**0.15** [131]
Quality of Life [133]	13-item measure given to participant and study partner regarding perceived quality of life of participant.	NA
Alzheimer's Disease Cooperative Study — Activities of Daily Living [83]	Measure given to study partner regarding independence in basic and instrumental activities of daily living.	NA
Epworth Sleepiness Scale [84]	Rating of likelihood of falling asleep during activities, scored 0-24	**0.47** [94]
Resource Utilization in Dementia — Lite[82,132]	Survey of heath care resource use and time for providing participant care completed by study partner.	NA

NA — not available.
Note: All citations listed by number are not contained in any of the prior reviews listed.
[a] UDS Battery Subtest.
[b] Active Battery Subtest (or close analogue).

2.5. Randomization and study blinding

Participants are randomized equally to either aerobic or non-aerobic exercise. A block randomization procedure is used, stratified by age (<75 years vs. >=75 years old) and gender to ensure the groups are well-matched across these variables. The randomization sequences were constructed prior to study start by the KU Department of Biostatistics. Randomization is performed immediately upon successful completion of the exercise test.

The psychometrician, clinical evaluator, and staff involved in the exercise testing are blinded to randomization assignment. Participants are asked at the beginning of each visit not to discuss anything regarding their intervention with testing staff. However, maintaining blinding can be a challenge with cognitively impaired participants. The PI remains un-blinded to perform safety assessments, review laboratory data, and address safety concerns or adverse events. Participants are not blinded to intervention arms. However, at the time of consent, the phrases “aerobic exercise” and “non-aerobic activities” are used rather than using “intervention” and “control”.

2.6. Testing considerations

Cognitive and physical function testing is generally performed by a single, trained rater from a prepared script and

study-specific instruction manual. In the rare instances that the primary tester is unavailable, a second rater who has trained with the primary rater administers tests from the same script and instruction manual. The rater who administered baseline measures, tests the participant throughout the study to maintain consistency. Though cognitive tests are not used in dementia staging process, tests and measures are discussed at the diagnostic consensus conference attended by the evaluating clinicians, a clinical neuropsychologist investigator and a physical therapist investigator and questions or concerns can be raised at this time.

2.7. Exercise intervention justification

We suspect that walking at moderate intensity is the most common form of exercise for older adults, is easily adopted and widely prescribed. Thus, it is likely easily adopted by this population. The current study design attempts to isolate aerobic exercise in order to examine its role in enhancing cognition. The intervention length of 26 weeks was chosen as substantial physiological adaptations to exercise occur in the first 3 to 4 months of exercise and major changes are unlikely beyond 6 months of training [66]. Considering the increased logistical and participant burden of longer durations, we chose a 26-week intervention for this study.

2.8. Exercise intervention implementation

All participants follow the same protocol with regard to warm up and cool down, duration of exercise session, one-on-one trainer time as well as for heart rate monitoring during the exercise session (Polar F4 or FT4, Polar Electro Inc., Lake Success, NY). The study coordinator meets with each participant at the Y to review randomization and facilitate an introduction to the assigned certified personal trainer (CPT) and facility. At that time participants are provided an exercise log and a Polar heart rate monitor, which are kept at the Y in a provided study box typically in the CPT's locked office. The exercise log contains 26 sheets and includes a place to record AEs as well as a weekly goal for duration and intensity (aerobic arm) or duration and activity (control group). All participants begin the intervention with a weekly goal of 60 min in Week 1 and increase their weekly exercise duration goals by approximately 21 min per week until they achieve the current public health recommended target duration of 150 min per week. Participants are asked to accomplish this target duration in 3–5 sessions so that sessions are no less than 30 min and no more than 50 min per day. Total exercise duration, duration in the target zone, average heart rate, peak heart rate, activity and a rating of perceived exertion (Borg 6–20) are gathered during each exercise session. CPTs and study staff assist participants with Polar monitors, how to keep their heart rate within a target zone and how to retrieve the session data from the watch to record on the exercise log. Data are recorded at the end of every session. If the participant does not become independent with retrieving data from the watch, the CPT is instructed to do so every session for the participant.

Participants are directly supervised by the CPT during all exercise sessions for the first 6 weeks of exercise after which direct supervision occurs during at least one session a week. We encourage each Y facility to have 2 or more CPTs trained and competent to lead both arms of our study protocol. This is intended to provide increased convenience for the participant while maintaining direct contact to enhance adherence. During supervised sessions, CPTs check in with participants to assess AEs, review exercise logs and encourage continued participation. The study coordinator and the CPT make a decision as to whether the individual is ready to exercise independently. The decision is based on the ability to use the heart rate monitor and gather information from the watch, safety and adherence to the exercise routine. If participants are unable or unwilling to use the treadmill, CPTs educate them on the non-weight bearing modalities such as a recumbent stepper or stationary bicycle.

The study pays for a household membership to the Y for the duration of the intervention as well as the personal trainer fees. All exercise sessions are completed at the Y. The aerobic arm utilizes Y treadmills and the non-aerobic arm utilizes Y equipment for the various activities. The study provides the resistance bands in to standardize this activity. While the CPT is training a study participant, he/she is considered a research assistant on the project and therefore reports to the study coordinator with regard to study-related activities. The study coordinator meets with each participant at the Y to review randomization and facilitate an introduction to the assigned certified personal trainer (CPT) and facility.

2.9. Aerobic exercise arm

Target heart rate (THR) zones are calculated for each participant randomized to the aerobic exercise arm. Both exercise duration and THR zone are manipulated to gradually introduce exercise and maximize aerobic training benefit. Participants are instructed to gradually increase speed and incline on the treadmill over approximately 5 min to warm-up and gradually reach their THR. The first 4 weeks of exercise, the THR zone is 40–55% of heart rate reserve (HRR) as calculated by the Karvonen formula from resting and peak heart rate gathered during the exercise test. The THR zone for Weeks 5–18 is 50–65 % of HRR. The THR for the final 8 weeks is 60–75% of HRR. Participants are instructed to gradually decrease speed and incline on the treadmill over approximately 5 min to cool down. Participants who cannot tolerate treadmill are instructed on use of an alternative aerobic modality.

2.10. Non-aerobic activities arm

Stretching and toning exercises such as those in our non-aerobic activities arm are commonly used as a control condition in research studies of aerobic exercise [87–89]. Participants in the non-aerobic activities group are given a THR of <100 bpm. A pictorial manual of activities is provided to each CPT and the participants. The activities include axial and appendicular stretching and toning exercises, seated exercises on a large diameter exercise ball and modified versions of Tai Chi and yoga. All non-aerobic exercise session begin with a slow-paced walk on the track for approximately 5 min to provide a warm-up for the participant. CPTs are provided with a schedule of specific exercises and verbal

instructions to participants. CPTs are encouraged to be creative with these activities as long as the variations fall within the intent of the exercises in the manual, are not progressive and do not cause heart rate to surpass the THR. All non-aerobic exercise sessions end with stretching exercises to provide a cool-down for the participant.

2.11. Community implementation

Successful delivery of an exercise program to a large community such as the Kansas City metropolitan area requires several easily accessible sites. The KU ADC established a partnership with the Y because of its significant infrastructure (18 locations), brand recognition within the aging community, and long-time association with physical activity and health promotion. The synergistic relationship between the KU ADC and the Y is at the core of the study. The key on which the partnership was built was agreement that the study results will inform methods in which community organizations such as the Y can serve older adults with memory loss. Importantly, the ADEPT research objectives match the mission and vision of the Y to serve the community through supporting healthy living. The KU ADC expands the Y's capacity to serve older adults and broadens their scope of activities beyond traditional membership-based exercise programming. Working with the KU ADC enhances the Y's ability to provide healthy living opportunities to the community in innovative ways. In return, the KU ADC receives the dedication of and access to facilities and CPTs. The network of 18 facilities expands the reach of the study and eases participant time and travel burden, and in many cases introduces individuals to a neighborhood resource they have never used.

As with any partnership between institutions with different policies there are challenges to overcome. First, training for research-level human subjects' protections is not standard for the Y and second, Y staff members bring various levels of familiarity with clinical trial methods as well as with aging and memory loss. Unique mechanisms for trainer time and membership reimbursement have also been developed to accommodate the study. The study coordinator works with the Business Director of Corporate Billing at the Y central office to direct bill for each study membership and CPT hours spent training our participants. The study coordinator communicates the required information for membership set-up and the Business Director sets up the membership remotely in the appropriate Y's database ready for the participant's arrival. The CPTs track their contact time with participants on a provided ADEPT time sheet and submit the time sheet to the Business Director to invoice us for those hours. The study coordinator verifies the trainer hours with the exercise logs.

To standardize implementation of the intervention protocols and facilitate training of CPTs, a study training manual was developed by KU ADC staff. All CPTs who work with study participants receive the study training manual as well as extensive in-person training by the study coordinator. The study training manual contains detailed instruction on the study protocol, maintaining adherence to the study protocol, reporting AEs, recording study data, the importance of human subjects' protection as well as instruction on working with people with dementia. The importance of the trainer to follow the protocol and to be the "eyes-and-ears" of the study is heavily emphasized. Federal and institutionally required human subjects' protection and HIPAA compliance courses are completed by CPTs through online training modules hosted by the University of Kansas Medical Center.

2.12. Adverse events

The study coordinator assesses AEs within 3 days of initiating exercise intervention and the CPTs are asked to assess for AEs or medication changes at the beginning of each visit. When the CPT is not supervising the participant, participants are instructed to note the AE on their exercise log as well as report the AE to the study coordinator by phone. CPTs are provided a standard form modified from previous community-based studies [90] to assist with identifying and documenting AEs. These documents are faxed to the KU ADC, at which time a follow-up call is made to the participant to obtain additional information regarding the AE. All AEs and medication changes from baseline are logged and entered in to the study database. Participants and their study partner are contacted by phone by the study coordinator for formal review of medication changes, medical history, and AEs at weeks 3, 6, 9, 16, 19, and 22. The seriousness of the AE is determined using the National Cancer Institute Common Terminology Criteria for Adverse Events v3.0. If the participant, study partner, or CPT reports AEs or complaints to the study coordinator, the AE is evaluated by the unblinded investigator and a course of action is determined. CPTs are instructed to follow the Y's emergency response protocol in the event of such emergency and to follow up by contacting the study coordinator.

2.13. Retention and adherence

The periodic telephone assessments by the study coordinator complement the CPTs routine daily assessment of AEs as well as enhance communication between the study team and the participants. Additionally, the calls encourage compliance with the intervention. To further enhance compliance, the study coordinator visits the facilities bi-weekly when study participants are exercising. This visit provides an opportunity to ensure protocol adherence by reviewing the logs for compliance, providing feedback and additional training on the protocol and stressing the importance of complying with the study protocol to both CPTs and participants. Copies of the exercise logs are collected for staff review and data entry. If the study coordinator does not make contact with a study participant during the visit, a message is written on the current week's exercise log. These messages are meant to inform the participant the study coordinator came to visit; to provide words of encouragement; and relay any relevant notes about the participant's progress.

2.14. Sample size and power calculation

Because this is a pilot study, the sample size was determined to provide estimates of intervention effect sizes on outcome measures. However, the moderate sample size is sufficient for preliminary hypothesis testing. We are recruiting 80 patients with the expectation that 60 individuals will

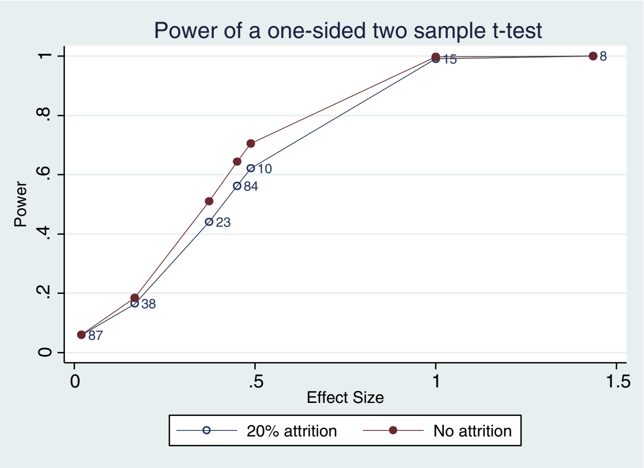

Fig. 1. The power of our study at testing different effect sizes using one-sided two sample t-test at alpha level of .05 when the attrition rate is 20% or 0. The observed effect sizes of Logic Memory from the 7 studies in the literature are marked in the plot. Each number on the plot denotes the sample size of the corresponding study. From this plot, a reasonable estimate of the effect size of logic memory is about .45 and the power is within .56 to .64 depending on the attrition rate. The power of the study is from .64 to .72 if the effect size is .5.

eventually complete the study, a sample size sufficient to plan a more definitive future study.

We also estimated the power of this study based on prior reports (see Table 3). We reviewed the exercise intervention literature in geriatric populations that used similar primary outcome measures to gain effect size estimates. Estimates obtained using AD patient populations and longitudinal designs were used preferentially. We found effect sizes for Logical Memory [91] (7 studies; effect size ranges from 0.03 to 1.44 and sample size ranges from 8 to 87). Fig. 1 shows the power of our study at testing different effect sizes reported in the literature using one-sided two sample t-test at alpha level of 0.05 when the attrition rate is 20% or 0. The power of the present study is between 0.64 and 0.72 if the effect size is 0.5. Cognitive and functional test data (primary outcome measures) are collected at baseline, Week 13 and Week 26 to enhance power as these are less directly associated with exercise compared to peak oxygen consumption or the Physical Performance Test for example. Currently, the study has excellent retention with only 2% drop out and approximately half of study participants enrolled.

2.15. Analysis plan

Using latent variable analysis we will aggregate common (true) score instrument items to generate the two co-primary outcomes for Aim 1: Verbal Memory and Executive Function. Latent variable analysis is a widely used statistical method to aggregate common variance across items and attenuate error. Latent variable analysis allows extraction of what is domain specific among several contributing items/tasks. Because this method pools common score variance and minimizes error variance (idiosyncratic to the item/task), resultant summary scores provide purer measurement of target domains than do individual tasks [92] Resultant summary scores yield more reliable, sensitive, and specific estimates of ability than do traditional analyses of raw scores or scaled score composites because they are purer indices of ability. Hypothesized causal relationships among these summary variables can be empirically tested using structural equation modeling.

We will use a linear mixed model to analyze each primary outcome measure. We will estimate the effect of intervention versus control as the interaction between group and time and also estimate the effect size of intervention using liner contrasts. We will test the linear trend difference between the exercise group and the control group. A linear mixed model is expected to be slightly more powerful than a t-test because of the repeated measures and the capability of the model at accommodating missing data. As this is a pilot study with small sample size, all the analysis will be conducted individually without adjustment for multiple outcomes.

Secondary measures will be evaluated in a similar manner as our primary outcomes. Secondary outcomes will be clearly indicated as such in resulting manuscripts.

Because of the attention paid to uniformly training CPTs we expect that training site have minimum impact on the outcomes, and anticipate that 10 of the 18 local Y sites for training and each site may provide 1–3 CPTs. With 60 patients in total, patients allocated to sites and CPTs will be too sparse for complicated hierarchical regression models. We will not take the cluster effects of Y site and trainer into analysis. ITT may be examined and estimated for future studies. Every reasonable effort is made to maintain rater consistency from baseline to follow-up.

VBM-indexed brain atrophy between intervention groups will be our exploratory neuroimaging outcome measure. The final tissue maps of gray matter, white matter and cerebrospinal fluid will be modulated in order to analyze relative volume differences between exercise groups and over time. This approach has been used effectively in exercise studies of similar duration in nondemented older adults [93].

We will determine circulating levels of metabolic and inflammatory biomarker levels (Insulin, IGF-1, IL-6, and TNFa). A blood draw will be performed following an overnight (~12 h) fast. Serum will be collected and assayed using commercially available ELISA assays (ALPCO Diagnostics).

3. Discussion

The purpose of this paper is to report our project protocol to guide future studies. While this study is too small to draw definitive conclusions, it may impact public health policy by encouraging research into the effects of aerobic exercise on AD. It may also stimulate development of community-deliverable aerobic exercise programs. The long-term objective of this research line is to assess the degree to which aerobic exercise is associated with healthy brain aging and reduced AD progression. The idea that regular exercise and physical activity has positive benefits for general health, reduces morbidity and mortality, and is an essential component of managing chronic disease is beyond discredit. However, it remains uncertain how exercise may benefit brain health and cognitive function in AD, now one of our most costly and pervasive age-related diseases.

ADEPT is designed to address previous study limitations that confound interpretation of aerobic effects on brain health and AD progression. Block randomization is performed to ensure balance of age and sex across treatment arms. Our control arm (stretching and toning) provides equal social contact and physical engagement without aerobic activity for participants. An additional improvement over prior studies is our careful characterization of potential participants and inclusion of only those in the earliest stages of AD. By using a standard evaluation, diagnosis and cognitive work up, we can be confident that our cohort reflects a group in the earliest stages of AD rather than the potentially mixed cohort of previous studies [41–51].

The partnership between the KU ADC and the Y provides additional advantages for conducting research. This arrangement provides evidence regarding translation of findings to a community deployable program. Additionally, conveniently located community facilities reduce participant and study partner burden. Y facilities employ CPTs and serve as a focal point for community engagement and participation. Older individuals tend to be familiar with the brand and in our experience, have been so positive about attending sessions at the Y they have purchased a membership after study participation. Key to a successful study is engagement of both the participant and study partner [94]. In ADEPT, as with previous studies [40] both the participant with AD and the study partner can engage in the exercise. This dyadic inclusion and focus may further improve study engagement and retention.

3.1. Limitations

As with any study, there are limitations inherent in the design. The community-based intervention increases the possibility that differences between CPTs will influence outcomes. To address this, the CPTs undergo extensive protocol training and we maintain a “hotline” phone number that is staffed at all times for trainer or participant questions. Maintaining open lines of communication with study staff allows CPTs to ask questions about the study protocol and report AEs. A second limitation of this study is its scope. The study is not large enough for definitive evaluation of aerobic exercise effects on AD. That being said, partnership with the Y offers a national infrastructure for deploying a more definitive future trial powered by the common and responsive outcome measures of cognition and function chosen for this study.

4. Conclusion

We have described a small pilot trial of aerobic versus non-aerobic exercise for those in the earliest stages of AD. The foundation of this study is the partnership of a public academic medical center and private enterprise to deliver the intervention in a familiar environment using readily available community resources. Scientific rigor is maintained while minimizing participant burden. The results will inform the development of a more definitive multi-site clinical trial that could be delivered in almost any city with a research university and community fitness resources.

Acknowledgments

The ADEPT study is supported by the National Institute on Aging (NIA) R01AG033673. Dr. Burns is also supported by grants from the NIA and NINDS (R01AG034614 and U10NS077356). Dr. Vidoni is supported in part by Frontiers: The Heartland Institute for Clinical and Translational Research (University of Kansas Medical Center's CTSA (KL2RR033177). Work conducted in the project is supported by the National Center for Research Resources (M01RR023940), and is now at the National Center for Advancing Translational Sciences (UL1RR033179). The content is solely the responsibility of the authors and does not necessarily represent the official views of the NIH. Drs. Burns, Vidoni, Anderson and Ms. Goodwin and Ms. Van Sciver are supported by the University of Kansas Alzheimer's Disease Center (P30AG035982). Specifically, Dir. Jon Mahnken of the Data Management & Statistics Core and Dir. Bill Brooks of the Neuroimaging Core of the KU ADC provided essential expertise. The KU Grayhawk Database provided contact information for potential participants.

We thank the staff of the YMCA of Greater Kansas City for their commitment to the study. The content is solely the responsibility of the authors and does not necessarily represent the official views of the NIH.

References

[1] Statistics FIFoA-R. Older americans 2008: key indicators of well-beingIn: Statistics FAFoA-R, editor. ; 2009.Washington, DC.
[2] U.S. Census Bureau. Population projections; 2008.
[3] National Institute on Aging. Growing older in america: the health and retirement study. In: Karp F, editor. Bethesda, MD: National Institutes of Health; 2007.
[4] Thies W, Bleiler L. Alzheimer's disease facts and figures. Alzheimers Dement 2011;7:208-44.
[5] Kawas CH, Katzman R. Epidemiology of dementia and Alzheimer disease. In: Terry R, Katzman J, Bick K, Sisodia S, editors. Alzheimer disease. 2nd ed. Philadelphia, PA: Lippincott Williams & Wilkins; 1999. p. 95–116.
[6] Nichol K, Deeny SP, Seif J, Camaclang K, Cotman CW. Exercise improves cognition and hippocampal plasticity in APOE epsilon4 mice. Alzheimers Dement 2009;5:287-94.
[7] Y. Garcia-Mesa, J.C. Lopez-Ramos, L. Gimenez-Llort, S. Revilla, R. Guerra, A. Gruart, et al. Physical exercise protects against Alzheimer's disease in 3xTg-AD mice. J Alzheimers Dis 24:421–54.
[8] van Praag H, Christie BR, Sejnowski TJ, Gage FH. Running enhances neurogenesis, learning, and long-term potentiation in mice. Proc Natl Acad Sci U S A 1999;96:13427-31.
[9] Barde YA. Neurotrophins: a family of proteins supporting the survival of neurons. Prog Clin Biol Res 1994;390:45-56.

[10] Stummer W, Weber K, Tranmer B, Baethmann A, Kempski O. Reduced mortality and brain damage after locomotor activity in gerbil forebrain ischemia. Stroke 1994;25:1862-9.
[11] Carro E, Trejo JL, Busiguina S, Torres-Aleman I. Circulating insulin-like growth factor I mediates the protective effects of physical exercise against brain insults of different etiology and anatomy. J Neurosci 2001;21:5678-84.
[12] Lu B, Chow A. Neurotrophins and hippocampal synaptic transmission and plasticity. J Neurosci Res 1999;58:76-87.
[13] Black JE, Isaacs KR, Anderson BJ, Alcantara AA, Greenough WT. Learning causes synaptogenesis, whereas motor activity causes angiogenesis, in cerebellar cortex of adult rats. Proc Natl Acad Sci U S A 1990;87:5568-72.
[14] Isaacs KR, Anderson BJ, Alcantara AA, Black JE, Greenough WT. Exercise and the brain: angiogenesis in the adult rat cerebellum after vigorous physical activity and motor skill learning. J Cereb Blood Flow Metab 1992;12:110-9.
[15] Cotman CW, Berchtold NC. Exercise: a behavioral intervention to enhance brain health and plasticity. Trends Neurosci 2002;25:295-301.
[16] R.M. Escorihuela, A. Tobena, A. Fernandez-Teruel. Environmental enrichment and postnatal handling prevent spatial learning deficits in aged hypoemotional (Roman high-avoidance) and hyperemotional (Roman low-avoidance) rats. Learn Mem (Cold Spring Harbor, NY) 2:40–8.
[17] Blair SN, Kampert JB, Kohl III HW, Barlow CE, Macera CA, Paffenbarger Jr RS, et al. Influences of cardiorespiratory fitness and other precursors on cardiovascular disease and all-cause mortality in men and women. JAMA 1996;276:205-10.
[18] Lakka TA, Laukkanen JA, Rauramaa R, Salonen R, Lakka HM, Kaplan GA, et al. Cardiorespiratory fitness and the progression of carotid atherosclerosis in middle-aged men. Ann Intern Med 2001;134:12-20.
[19] Kurl S, Laukkanen JA, Rauramaa R, Lakka TA, Sivenius J, Salonen JT. Cardiorespiratory fitness and the risk for stroke in men. Arch Intern Med 2003;163:1682-8.
[20] Seals DR, Hagberg JM, Hurley BF, Ehsani AA, Holloszy JO. Effects of endurance training on glucose tolerance and plasma lipid levels in older men and women. JAMA 1984;252:645-9.
[21] Hughes VA, Fiatarone MA, Fielding RA, Kahn BB, Ferrara CM, Shepherd P, et al. Exercise increases muscle GLUT-4 levels and insulin action in subjects with impaired glucose tolerance. Am J Physiol 1993;264:E855-62.
[22] Cox JH, Cortright RN, Dohm GL, Houmard JA. Effect of aging on response to exercise training in humans: skeletal muscle GLUT-4 and insulin sensitivity. J Appl Physiol 1999;86:2019-25.
[23] Kahn SE, Larson VG, Beard JC, Cain KC, Fellingham GW, Schwartz RS, et al. Effect of exercise on insulin action, glucose tolerance, and insulin secretion in aging. Am J Physiol 1990;258:E937-43.
[24] Houmard JA, Tyndall GL, Midyette JB, Hickey MS, Dolan PL, Gavigan KE, et al. Effect of reduced training and training cessation on insulin action and muscle GLUT-4. J Appl Physiol 1996;81:1162-8.
[25] Gustafson D, Rothenberg E, Blennow K, Steen B, Skoog I. An 18-year follow-up of overweight and risk of Alzheimer disease. Arch Intern Med 2003;163:1524-8.
[26] Adlard PA, Perreau VM, Pop V, Cotman CW. Voluntary exercise decreases amyloid load in a transgenic model of Alzheimer's disease. J Neurosci 2005;25:4217-21.
[27] Wu CW, Chang YT, Yu L, Chen HI, Jen CJ, Wu SY, et al. Exercise enhances the proliferation of neural stem cells and neurite growth and survival of neuronal progenitor cells in dentate gyrus of middle-aged mice. J Appl Physiol 2008;105:1585-94.
[28] Littbrand H, Stenvall M, Rosendahl E. Applicability and effects of physical exercise on physical and cognitive functions and activities of daily living among people with dementia: a systematic review. Am J Phys Med Rehabil 2011;90:495-518.
[29] Daviglus ML, Bell CC, Berrettini W, Bowen PE, Connolly Jr ES, Cox NJ, et al. National Institutes of Health State-of-the-Science Conference statement: preventing Alzheimer disease and cognitive decline. Ann Intern Med 2010;153:176-81.
[30] O'Connor S, Prusiner SB, Dychtwald K. The age of Alzheimer's. The New York Time. New York: Times Co.; 2010. p. A33.
[31] Heyn P, Abreu BC, Ottenbacher KJ. The effects of exercise training on elderly persons with cognitive impairment and dementia: a meta-analysis. Arch Phys Med Rehabil 2004;85:1694-704.
[32] Venturelli M, Scarsini R, Schena F. Six-month walking program changes cognitive and ADL performance in patients with Alzheimer. Am J Alzheimers Dis Other Demen 2011;26:381-8.
[33] Williams CL, Tappen RM. Exercise training for depressed older adults with Alzheimer's disease. Aging Ment Health 2008;12:72-80.
[34] Rolland Y, Pillard F, Klapouszczak A, Reynish E, Thomas D, Andrieu S, et al. Exercise program for nursing home residents with Alzheimer's disease: a 1-year randomized, controlled trial. J Am Geriatr Soc 2007;55:158-65.
[35] Landi F, Russo A, Bernabei R. Physical activity and behavior in the elderly: a pilot study. Arch Gerontol Geriatr Suppl 2004:235-41.
[36] Santana-Sosa E, Barriopedro MI, Lopez-Mojares LM, Perez M, Lucia A. Exercise training is beneficial for Alzheimer's patients. Int J Sports Med 2008;29:845-50.
[37] Kemoun G, Thibaud M, Roumagne N, Carette P, Albinet C, Toussaint L, et al. Effects of a physical training programme on cognitive function and walking efficiency in elderly persons with dementia. Dement Geriatr Cogn Disord 2010;29:109-14.
[38] Hauer K, Schwenk M, Zieschang T, Essig M, Becker C, Oster P. Physical training improves motor performance in people with dementia: a randomized controlled trial. J Am Geriatr Soc 2012;60:8–15.
[39] Lautenschlager NT, Cox KL, Flicker L, Foster JK, van Bockxmeer FM, Xiao J, et al. Effect of physical activity on cognitive function in older adults at risk for Alzheimer disease: a randomized trial. JAMA 2008;300:1027-37.
[40] Teri L, Gibbons LE, McCurry SM, Logsdon RG, Buchner DM, Barlow WE, et al. Exercise plus behavioral management in patients with Alzheimer disease: a randomized controlled trial. JAMA 2003;290:2015-22.
[41] McMurdo M, Rennie L. Improvements in quadriceps strength with regular seated exercise in the institutionalized elderly. Arch Phys Med Rehabil 1994;75:600-3.
[42] Fiatarone M, O'Neill EF, Ryan ND, Clements KM, Solares GR, Nelson ME, et al. Exercise training and nutritional supplementation for physical frailty in very elderly people. [see comment]N Engl J Med 1994;330:1769-75.
[43] Meuleman JR, Brechue WF, Kubilis PS, Lowenthal DT. Exercise training in the debilitated aged: strength and functional outcomes. Arch Phys Med Rehabil 2000;81:312-8.
[44] Lazowski DA, Ecclestone NA, Myers AM, Paterson DH, Tudor-Locke C, Fitzgerald C, et al. A randomized outcome evaluation of group exercise programs in long-term care institutions. J Gerontol A Biol Sci Med Sci 1999;54:M621-8.
[45] McMurdo ME, Rennie L. A controlled trial of exercise by residents of old people's homes. Age Ageing 1993;22:11-5.
[46] Tappen RM, Roach KE, Applegate EB, Stowell P. Effect of a combined walking and conversation intervention on functional mobility of nursing home residents with Alzheimer disease. Alzheimer Dis Assoc Disord 2000;14:196-201.
[47] Nowalk MP, Prendergast JM, Bayles CM, Amico FJ, Colvin GC. A randomized trial of exercise programs among older individuals living in two long-term care facilities: the FallsFREE program. J Am Geriatr Soc 2001;49:859-65.
[48] Schnelle JF, MacRae PG, Ouslander JG, Simmons SF, Nitta M. Functional incidental training, mobility performance, and incontinence care with nursing home residents. [see comment]J Am Geriatr Soc 1995;43:1356-62.
[49] Schnelle J, MacRae PG, Giacobassi K, MacRae HS, Simmons SF, Ouslander J. Exercise with physically restrained nursing home residents: maximizing benefits of restraint reduction. J Am Geriatr Soc 1996;44:507-12.
[50] Alessi. Does physical activity improve sleep in impaired nursing home residents? J Am Geriatr Soc 1995;43:1098-102.
[51] Pomeroy VM, Warren CM, Honeycombe C, Briggs RS, Wilkinson DG, Pickering RM, et al. Mobility and dementia: is physiotherapy treatment during respite care effective? Int J Geriatr Psychiatry 1999;14:389-97.
[52] Roach KE, Tappen RM, Kirk-Sanchez N, Williams CL, Loewenstein D. A randomized controlled trial of an activity specific exercise program for individuals with Alzheimer disease in long-term care settings. J Geriatr Phys Ther 2011;34:50-6.
[53] Miller LA, Spitznagel MB, Busko S, Potter V, Juvancic-Heltzel J, Istenes N, et al. Structured exercise does not stabilize cognitive function in individuals with mild cognitive impairment residing in a structured living facility. Int J Neurosci 2011;121:218-23.
[54] Arkin SM. Elder rehab: a student-supervised exercise program for Alzheimer's patients. Gerontologist 1999;39:729-35.
[55] Baker LD, Frank LL, Foster-Schubert K, Green PS, Wilkinson CW, McTiernan A, et al. Effects of aerobic exercise on mild cognitive impairment: a controlled trial. Arch Neurol 2010;67:71-9.
[56] Alessi CA, Yoon EJ, Schnelle JF, Al-Samarrai NR, Cruise PA. A randomized trial of a combined physical activity and environmental intervention in nursing home residents: do sleep and agitation improve? J Am Geriatr Soc 1999;47:784-91.
[57] Chandler JM, Duncan PW, Kochersberger MD, Studenski S. Is lower extremity strength gain associated with improvement in physical performance and disability in frail, community-dwelling elders? Arch Phys Med Rehabil 1998;79:24-30.
[58] Vreugdenhil A, Cannell J, Davies A, Razay G. A community-based exercise programme to improve functional ability in people with Alzheimer's disease: a randomized controlled trial. Scand J Caring Sci 2012;26:12-9.
[59] Steinberg M, Leoutsakos JM, Podewils LJ, Lyketsos CG. Evaluation of a home-based exercise program in the treatment of Alzheimer's disease: the Maximizing Independence in Dementia (MIND) study. Int J Geriatr Psychiatry 2009;24:680-5.

[60] Burns JM, Cronk BB, Anderson HS, Donnelly JE, Thomas GP, Harsha A, et al. Cardiorespiratory fitness and brain atrophy in early Alzheimer disease. Neurology 2008;71:210-6.
[61] Honea RA, Thomas GP, Harsha A, Anderson HS, Donnelly JE, Brooks WM, et al. Cardiorespiratory fitness and preserved medial temporal lobe volume in Alzheimer disease. Alzheimer Dis Assoc Disord 2009;23: 188-97.
[62] Vidoni ED, Honea RA, Billinger SA, Swerdlow RH, Burns JM. Cardiorespiratory fitness is associated with atrophy in Alzheimer's and aging over 2 years. Neurobiol Aging 2012;33:1624-32.
[63] Ahlskog JE, Geda YE, Graff-Radford NR, Petersen RC. Physical exercise as a preventive or disease-modifying treatment of dementia and brain aging. Mayo Clin Proc 2011;86:876-84.
[64] Burns JM, Mayo MS, Anderson HS, Smith HJ, Donnelly JE. Cardiorespiratory fitness in early-stage Alzheimer disease. Alzheimer Dis Assoc Disord 2008;22:39-46.
[65] Nelson ME, Rejeski WJ, Blair SN, Duncan PW, Judge JO, King AC, et al. Physical activity and public health in older adults: recommendation from the American College of Sports Medicine and the American Heart Association. Med Sci Sports Exerc 2007;39:1435-45.
[66] Morss GM, Jordan AN, Skinner JS, Dunn AL, Church TS, Earnest CP, et al. Dose response to exercise in women aged 45–75 yr (DREW): design and rationale. Med Sci Sports Exerc 2004;36:336-44.
[67] McKhann GM, Knopman DS, Chertkow H, Hyman BT, Jack Jr CR, Kawas CH, et al. The diagnosis of dementia due to Alzheimer's disease: recommendations from the National Institute on Aging-Alzheimer's Association workgroups on diagnostic guidelines for Alzheimer's disease. Alzheimers Dement 2011;7:263-9.
[68] Morris JC, Storandt M, Miller JP, McKeel DW, Price JL, Rubin EH, et al. Mild cognitive impairment represents early-stage Alzheimer disease. Arch Neurol 2001;58:397-405.
[69] Albert MS, DeKosky ST, Dickson D, Dubois B, Feldman HH, Fox NC, et al. The diagnosis of mild cognitive impairment due to Alzheimer's disease: recommendations from the National Institute on Aging-Alzheimer's Association workgroups on diagnostic guidelines for Alzheimer's disease. Alzheimers Dement 2011;7:270-9.
[70] Billinger SA, Vidoni ED, Honea RA, Burns JM. Cardiorespiratory response to exercise testing in individuals with Alzheimer's disease. Arch Phys Med Rehabil 2011;92:2000-5.
[71] Anderson HS, Kluding PM, Gajewski BJ, Donnelly JE, Burns JM. Reliability of peak treadmill exercise tests in mild Alzheimer disease. Int J Neurosci 2011;121:450-6.
[72] Gibbons RJ, Balady GJ, Beasley JW, Bricker JT, Duvernoy WF, Froelicher VF, et al. ACC/AHA Guidelines for Exercise Testing. A report of the American College of Cardiology/American Heart Association Task Force on Practice Guidelines (Committee on Exercise Testing). J Am Coll Cardiol 1997;30:260-311.
[73] Billinger SA, Tseng BY, Kluding PM. Modified total-body recumbent stepper exercise test for assessing peak oxygen consumption in people with chronic stroke. Phys Ther 2008;88:1188-95.
[74] Johnson DK, Storandt M, Morris JC, Langford ZD, Galvin JE. Cognitive profiles in dementia Alzheimer disease vs healthy brain aging. Neurology 2008;71:1783-9.
[75] Ownby RL, Matthews CG. On the meaning of the WISC-R third factor: relations to selected neuropsychological measures. J Consult Clin Psychol 1985;53:531-4.
[76] Salthouse TA, Ferrer-Caja E. What needs to be explained to account for age-related effects on multiple cognitive variables? Psychol Aging 2003;18:91–110.
[77] Zelinski EM, Lewis KL. Adult age differences in multiple cognitive functions: differentiation, dedifferentiation, or process-specific change? Psychol Aging 2003;18:727-45.
[78] Galvin JE, Johnson DK, Morris JC. Cognitive profiles of Parkinson's disease and Lewy body demetias. National Institute of Aging, Alzheimer's Disease Center Directors' Meeting; 2008.
[79] Logsdon RG, Gibbons LE, McCurry SM, Teri L. Assessing quality of life in older adults with cognitive impairment. Psychosom Med 2002;64: 510-9.
[80] Alexopoulos GS, Abrams RC, Young RC, Shamoian CA. Cornell scale for depression in dementia. Biol Psychiatry 1988;23:271-84.
[81] Feldman H, Sauter A, Donald A, Gelinas I, Gauthier S, Torfs K, et al. The disability assessment for dementia scale: a 12-month study of functional ability in mild to moderate severity Alzheimer disease. Alzheimer Dis Assoc Disord 2001;15:89-95.
[82] Wimo A, Jonsson L, Zbrozek A. The Resource Utilization in Dementia (RUD) instrument is valid for assessing informal care time in community-living patients with dementia. J Nutr Health Aging 2010;14:685-90.
[83] Galasko D, Bennett D, Sano M, Ernesto C, Thomas R, Grundman M, et al. An inventory to assess activities of daily living for clinical trials in Alzheimer's disease. The Alzheimer's Disease Cooperative Study. Alzheimer Dis Assoc Disord 1997(Suppl. 2):S33-9 [1997;11:S33-9].
[84] Johns MW. A new method for measuring daytime sleepiness: the Epworth sleepiness scale. Sleep 1991;14:540-5.
[85] Shah KR, Carr D, Roe CM, Miller JP, Coats M, Morris JC. Impaired physical performance and the assessment of dementia of the Alzheimer type. Alzheimer Dis Assoc Disord 2004;18:112-8.
[86] Rikli R, Jones J. Development and validation of a functional fitness test for community-residing older adults. J Aging Phys Act 1999;7:129-61.
[87] Kramer AF, Hahn S, Cohen NJ, Banich MT, McAuley E, Harrison CR, et al. Ageing, fitness and neurocognitive function. Nature 1999;400: 418-9.
[88] Colcombe SJ, Kramer AF, Erickson KI, Scalf P, McAuley E, Cohen NJ, et al. Cardiovascular fitness, cortical plasticity, and aging. Proc Natl Acad Sci U S A 2004;101:3316-21.
[89] Erickson KI, Raji CA, Lopez OL, Becker JT, Rosano C, Newman AB, et al. Physical activity predicts gray matter volume in late adulthood: The Cardiovascular Health Study. Neurology 2010;75:1415-22.
[90] Goodrich DE, Larkin AR, Lowery JC, Holleman RG, Richardson CR. Adverse events among high-risk participants in a home-based walking study: a descriptive study. Int J Behav Nutr Phys Act 2007;4:20.
[91] Wechsler D. Wechsler Memory Scale III: Administration and scoring manual. Orlando, FL: Psychological Corporation; 1997.
[92] Bollen KA. Structural equations with latent variables. New York: Wiley; 1989.
[93] Colcombe SJ, Erickson KI, Scalf PE, Kim JS, Prakash R, McAuley E, et al. Aerobic exercise training increases brain volume in aging humans. J Gerontol A Biol Sci Med Sci 2006;61:1166-70.
[94] Logsdon RG, McCurry SM, Teri L. A home health care approach to exercise for persons with Alzheimer's disease. Care Manag J 2005;6: 90-7.
[95] Mayer CJ, Steinman L, Williams B, Topolski TD, LoGerfo J. Developing a Telephone Assessment of Physical Activity (TAPA) questionnaire for older adults. Prev Chronic Dis 2008;5:A24.
[96] Grober E, Buschke H, Crystal H, Bang S, Dresner R. Screening for dementia by memory testing. Neurology 1988;38:900-3.
[97] Blumenthal JA, Emery CF, Madden DJ, Schniebolk S, Walsh-Riddle M, George LK, et al. Long-term effects of exercise on psychological functioning in older men and women. J Gerontol 1991;46:P352-61.
[98] Prakash RS, Snook EM, Erickson KI, Colcombe SJ, Voss MW, Motl RW, et al. Cardiorespiratory fitness: a predictor of cortical plasticity in multiple sclerosis. Neuroimage 2007;34:1238-44.
[99] Wechsler D. Administration and scoring manual: Wechsler Memory Scale. San Antonio, TX: Harcourt Brace; 1997.
[100] Fabre C, Chamari K, Mucci P, Masse-Biron J, Prefaut C. Improvement of cognitive function by mental and/or individualized aerobic training in healthy elderly subjects. Int J Sports Med 2002;23:415-21.
[101] Hill RD, Storandt M, Malley M. The impact of long-term exercise training on psychological function in older adults. J Gerontol 1993;48: P12-7.
[102] Khatri P, Blumenthal JA, Babyak MA, Craighead WE, Herman S, Baldewicz T, et al. Effects of exercise training an cognitive functioning among depressed older men and women. J Aging Phys Act 2001;9: 43-57.
[103] Molloy DW, Beerschoten DA, Borrie MJ, Crilly RG, Cape RD. Acute effects of exercise on neuropsychological function in elderly subjects. J Am Geriatr Soc 1988;36:29-33.
[104] Molloy DW, Richardson LD, Crilly RG. The effects of a three-month exercise programme on neuropsychological function in elderly institutionalized women: a randomized controlled trial. Age Ageing 1988;17:303-10.
[105] Powell RR. Psychological effects of exercise therapy upon institutionalized geriatric mental-patients. J Gerontol 1974;29:157-61.
[106] Shay KA, Roth DL. Association between aerobic fitness and visuospatial performance in healthy older adults. Psychol Aging 1992;7:15-24.
[107] Goodglass H, Kaplan E. The assessment of aphasia and related disorders. Philadelphia: Lea & Febiger; 1983.
[108] Blumenthal JA, Emery CF, Madden DJ, George LK, Coleman RE, Riddle MW, et al. Cardiovascular and behavioral effects of aerobic exercise training in healthy older men and women. J Gerontol 1989;44:M147-57.
[109] Dustman RE, Emmerson RY, Ruhling RO, Shearer DE, Steinhaus LA, Johnson SC, et al. Age and fitness effects on EEG, ERPs, visual sensitivity, and cognition. Neurobiol Aging 1990;11:193-200.
[110] Dustman RE, Ruhling RO, Russell EM, Shearer DE, Bonekat HW, Shigeoka JW, et al. Aerobic exercise training and improved neuropsychological function of older individuals. Neurobiol Aging 1984;5: 35-42.
[111] Barry AJ, Steinmetz JR, Page HF, Rodahl K. The effects of physical conditioning on older individuals. II. Motor performance and cognitive function. J Gerontol 1966;21:192-9.

[112] Gordon BA, Rykhlevskaia EI, Brumback CR, Lee Y, Elavsky S, Konopack JF, et al. Neuroanatomical correlates of aging, cardiopulmonary fitness level, and education. Psychophysiology 2008;45:825-38.
[113] Goodglass H, Kaplan E. Boston diagnostic apahsia examination. Philadelphia: Lea & Febiger; 1983.
[114] Emery CF, Schein RL, Hauck ER, MacIntyre NR. Psychological and cognitive outcomes of a randomized trial of exercise among patients with chronic obstructive pulmonary disease. Health Psychol 1998;17:232-40.
[115] Emery CF, Shermer RL, Hauck ER, Hsiao ET, MacIntyre NR. Cognitive and psychological outcomes of exercise in a 1-year follow-up study of patients with chronic obstructive pulmonary disease. Health Psychol 2003;22:598-604.
[116] El-Naggar AM. Physical training effect on relationship of physical, mental, and emotional fitness in adult men. J Hum Ergol (Tokyo) 1986;15:79-84.
[117] Emery CF. Effects of age on physiological and psychological functioning among COPD patients in an exercise program. J Aging Health 1994;6:3–16.
[118] Emery CF, Gatz M. Psychological and cognitive effects of an exercise program for community-residing older adults. Gerontologist 1990;30:184-8.
[119] Hassmen P, Koivula N. Mood, physical working capacity and cognitive performance in the elderly as related to physical activity. Aging (Milano) 1997;9:136-42.
[120] Hassmen P, Ceci R, Backman L. Exercise for older women: a training method and its influences on physical and cognitive performance. Eur J Appl Physiol Occup Physiol 1992;64:460-6.
[121] Ismail AH, El-Naggar AM. Effect of exercise on cognitive processing in adult men. J Hum Ergol (Tokyo) 1981;10:83-91.
[122] Izquierdo-Porrera A, Waldstein SR. Cardiovascular risk factors and cognitive function in African Americans. J Gerontol 2002;57B:P377.
[123] Williams P, Lord SR. Effects of group exercise on cognitive functioning and mood in older women. Aust N Z J Public Health 1997;21:45-52.
[124] Wechsler D. Adminstration and scoring manual: Wechsler Adult Intelligence Scale. San Antonio, TX: Harcourt Brace; 1997.
[125] Madden DJ, Blumenthal JA. Interaction of hypertension and age in visual selective attention performance. Health Psychol 1998;17:76-83.
[126] Armitage SG. An analysis of certain psychological tests used in the evaluation of brain injury. Psychol Monogr 1946;60:1–48.
[127] Thurstone LL, Thurstone LG. Examiner manual for the SRA primary mental abilities test. Chicago: Science Research Associates; 1949.
[128] Gauthier S, Gélinas I, Gauthier L. Functional disability in Alzheimer's disease. Int Psychogeriatr 1997;9:163-5.
[129] Behl P, Lanctot KL, Streiner DL, Guimont I, Black SE. Cholinesterase inhibitors slow decline in executive functions, rather than memory, in Alzheimer's disease: a 1-year observational study in the Sunnybrook dementia cohort. Curr Alzheimer Res 2006;3:147-56.
[130] Alexopoulos GS, Abrams RC, Young RC, Shamoian CA. Cornell scale for depression in dementia. Biol Psychiatry 1988;23:271-84.
[131] Khachaturian AS, Zandi PP, Lyketsos CG, Hayden KM, Skoog I, Norton MC, et al. Antihypertensive medication use and incident alzheimer disease: The Cache County Study. Arch Neurol 2006;20:93–100.
[132] Fratiglioni L, Viitanen M, Von Strauss E, Tontodonati V, Herlitz A, Winblad B. Very old women at highest risk of dementia and Alzheimer's disease: incidence data from the Kungsholmen Project, Stockholm. Neurology 1997;48:132-8.
[133] Logsdon RG, Gibbons LE, McCurry SM, Teri L. Assessing quality of life in older adults with cognitive impairment. Psychosom Med 2002;64:510-9.
